# P53 in human melanoma fails to regulate target genes associated with apoptosis and the cell cycle and may contribute to proliferation

**DOI:** 10.1186/1471-2407-11-203

**Published:** 2011-05-27

**Authors:** Kelly A Avery-Kiejda, Nikola A Bowden, Amanda J Croft, Lyndee L Scurr, Carla F Kairupan, Katie A Ashton, Bente A Talseth-Palmer, Helen Rizos, Xu D Zhang, Rodney J Scott, Peter Hersey

**Affiliations:** 1 Oncology and Immunology, Calvary Mater Newcastle Hospital, University of Newcastle, Newcastle, NSW, Australia; 2 Discipline of Medical Genetics, School of Biomedical Sciences, Faculty of Health, University of Newcastle at the Hunter Medical Research Institute, Newcastle, NSW, Australia; 3 Westmead Institute for Cancer Research, University of Sydney at Westmead Millennium Institute, Westmead Hospital, Westmead, NSW, Australia

## Abstract

**Background:**

Metastatic melanoma represents a major clinical problem. Its incidence continues to rise in western countries and there are currently no curative treatments. While mutation of the *P53 *tumour suppressor gene is a common feature of many types of cancer, mutational inactivation of *P53 *in melanoma is uncommon; however, its function often appears abnormal.

**Methods:**

In this study whole genome bead arrays were used to examine the transcript expression of P53 target genes in extracts from 82 melanoma metastases and 6 melanoma cell lines, to provide a global assessment of aberrant P53 function. The expression of these genes was also examined in extracts derived from diploid human melanocytes and fibroblasts.

**Results:**

The results indicated that P53 target transcripts involved in apoptosis were under-expressed in melanoma metastases and melanoma cell lines, while those involved in the cell cycle were over-expressed in melanoma cell lines. There was little difference in the transcript expression of P53 target genes between cell lines with null/mutant *P53 *compared to those with wild-type *P53*, suggesting that altered expression in melanoma was not related to *P53 *status. Similarly, down-regulation of P53 by short-hairpin RNA (shRNA) had limited effect on P53 target gene expression in melanoma cells, whereas there were a large number of P53 target genes whose mRNA expression was significantly altered by P53 inhibition in melanocytes. Analysis of whole genome gene expression profiles indicated that the ability of P53 to regulate genes involved in the cell cycle was significantly reduced in melanoma cells. Moreover, inhibition of P53 in melanocytes induced changes in gene expression profiles that were characteristic of melanoma cells and resulted in increased proliferation. Conversely, knockdown of P53 in melanoma cells resulted in decreased proliferation.

**Conclusions:**

These results indicate that P53 target genes involved in apoptosis and cell cycle regulation are aberrantly expressed in melanoma and that this aberrant functional activity of P53 may contribute to the proliferation of melanoma.

## Background

Metastatic melanoma represents a major clinical problem. The incidence of melanoma continues to rise in western countries, and because of its highly aggressive clinical behaviour and resistance to a wide range of therapies, there are currently no curative treatments once the disease spreads beyond locoregional sites [1-3]. While mutation of the *P53 *tumour suppressor gene is a common feature of many types of cancer [4], mutational inactivation of *P53 *in melanoma is uncommon and wild-type P53 is frequently expressed at high levels [5-9]. Moreover, unlike other cancers, the expression of wild-type P53 in melanoma appears to increase with tumour progression and depth of tumour invasion and is associated with worse prognostic features [5]. Thus, as judged from the malignant nature of melanoma and its unresponsiveness to available therapeutics including DNA-damaging agents [1], wild-type P53 in melanoma fails to function as a tumour suppressor.

In the normal cell, the tumour suppressor P53 plays a critical role in determining cell fate and has been classified as the "guardian of the genome". In response to genotoxic stress, P53 may promote either cell cycle arrest and DNA repair or apoptosis [10,11]. The outcome of P53 activation- life or death - is primarily due to its role in the transcriptional regulation of numerous genes involved in these responses [12,13]. High throughput chromatin immunoprecipitation (ChIP) analysis has estimated that P53 can bind to and potentially regulate the expression of around 500 to 1600 target genes [14,15], exemplifying its importance as a transcriptional regulator.

In human melanoma, P53 accumulates after genotoxic stress and retains its transcriptional activity, suggesting that signalling pathways upstream of P53 remain intact and that it is at least partly functional and can respond to stress [16-18]. However, it has also been reported that wild-type P53 may be aberrantly phosphorylated following ionising radiation (IR) and that there is a failure to promote cell cycle arrest or apoptosis, suggesting that signalling pathways downstream of P53 may be defective in melanoma [18]. A number of mechanisms for inhibition of P53 function in melanoma cells have been suggested, such as de-regulation of MDM2 and MDM4, over-expression of Y box-binding protein 1 (YB-1), loss of P53 adaptor proteins [19,20] and our own studies have suggested that P53 isoforms may be involved [16]. However, the exact P53 target genes - the ultimate effectors of P53 function - that become de-regulated in melanoma as a result of aberrant P53 signalling and allow it to bypass an apoptotic response, promoting resistance to treatment, remain to be elucidated.

In this study, the mRNA expression of known P53 target genes were examined in metastatic melanoma and melanoma cell lines and compared to normal cells using whole genome bead arrays. We report that a large proportion of P53 target genes, predominantly involved in apoptosis and cell cycle regulation, were significantly altered in metastatic melanoma and melanoma cell lines. Altered expression of these genes was not dependent on P53 status. Moreover, inhibition of P53 expression in melanoma cell lines had limited effect on P53 target gene expression, suggesting that constitutive regulation of P53 target gene expression is dampened in melanoma. Inhibition of P53 in melanocytes induced changes in P53 target gene expression that were characteristic of melanoma cells and resulted in increased proliferation. Conversely, knockdown of P53 in melanoma cells resulted in decreased proliferation. These results provide new information on the mRNA expression of P53-regulated target genes that become de-regulated in melanoma and that may contribute to the oncogenic process.

## Methods

### Melanoma samples

From February 2000 to December 2006, melanoma metastases were collected from 82 patients attending the Newcastle Melanoma Unit. The melanomas were cleaned of surrounding tissue, cut into 2-3 mm fragments and stored in vials in liquid nitrogen. Written consent was given by the patients for collection of their samples. This study complies with the Helsinki Declaration and was approved by the Hunter New England Health Research Ethics Committee (Approval No: 05/02/09/3.02). There were 40 females (mean age 61.6 ± 12.7 years) and 42 males (mean age 58.3 ± 15.1 years) in the study. The tissue was collected from the following sites in females: subcutaneous - 11, lymph nodes - 10, lung/liver - 7, bowel - 4, brain - 3, bone - 1 and occult - 4. The sites in males were: subcutaneous - 11, lymph nodes - 19, lung/liver - 9, bowel - 2 and occult - 1.

### Cell lines

The human melanoma cell lines Mel-RM, MM200, IgR3, Me1007, Me4405 and Sk-Mel-28 have been described previously [21]. Sk-Mel-28 had mutant *P53 *and Me4405 was null for *P53 *[16]. All melanoma cell lines were cultured in DMEM containing 5% FCS (Commonwealth Serum Laboratories, VIC, Australia) and maintained in exponential growth at 37°C and 5% CO_2_. Melanocytes were purchased from Cascade Biologics (OR, USA) and cultured in Medium 154 (Cascade Biologics). FLOW2000, WS-1 and HDF1314 fibroblasts were cultured in DMEM containing 10% FCS (Commonwealth Serum Laboratories).

### Stable transduction of cell lines

Short hairpin RNA (shRNA) sequences to *P53 *or a control were expressed in the *pSIH1-H1-copGFP *(Copepod green fluorescent protein) shRNA expression vector (Systems Biosciences, CA, USA). The *P53*-directed shRNA sequence corresponds to nucleotides 1026-1044 (Accession number NM_000546) [22]. The control shRNA sequence 5'-TTAGAGGCGAGCAAGACTA-3' showed no homology to any known human transcript. Lentiviruses were produced in HEK293T cells using the *pSIH1-H1-copGFP *shRNA expression vector (Systems Biosciences) encased in viral capsid encoded by three packaging plasmids as described previously [23]. Viruses were concentrated as described previously [24]. Viral titres were determined using 1 × 10^5 ^U2OS cells/well in 6-well plates, transduced with serial dilutions of the concentrated viral stocks in the presence of Polybrene (8 μg/ml; Sigma, NSW, Australia). Cells were harvested 48 hours post-transduction, analysed by flow cytometry for copGFP expression and viral titre calculated.

To generate P53 silenced stable cell lines, Mel-RM, IgR3 and melanocytes were transduced at an MOI of 10 with either a virus encoding *P53 *shRNA or control shRNA. Cells were transduced twice with three days in between each transduction. The efficiency of transduction was monitored with co-expression of copGFP and was consistently over 95%. All cell lines tested negative for the presence of replicative competent virus using the Retrotek HIV-1 p24 antigen ELISA kit (ZeptoMetrix Corporation, NY, USA).

### RNA extraction, amplification, labelling and hybridisation

Total RNA was extracted from melanoma cell lines, melanocytes and fibroblasts using the SV Total RNA Isolation System Kit according to the manufacturers' instructions (Promega, NSW, Australia) and from metastatic melanoma tissues (2-3 mm^2^) using Trizol reagent (Invitrogen, VIC, Australia) and the RNeasy Kit (Qiagen, VIC, Australia). The extracted RNA was amplified and biotinylated using the TotalPrep RNA Amplification kit according to the manufacturers' instructions (Ambion, TX, USA), then hybridised to Sentrix HumanRef-8 Expression Beadchips according to the manufacturers' instructions (Illumina, CA, USA). The arrays were scanned on a Bead Array Reader (Illumina).

### Microarray analysis

The expression of 20,589 transcripts was analysed in the metastatic melanoma and melanocyte cRNA samples using Illumina Sentrix HumanRef-8 Expression Beadchips (v2.0, Illumina). The expression of 24,526 transcripts was analysed in the cell line samples, using Illumina Sentrix HumanRef-8 Expression Beadchips (v3.0, Illumina). All samples were cubic spline normalised using BeadStudio 3.0 software (Illumina) and normalised to the median using GeneSpring GX v10.0 (Agilent Technologies, VIC, Australia). All subsequent analysis was performed using GeneSpring GX v10.0 (Agilent Technologies).

Two-hundred and ninety probes, representing 247 unique transcripts and 181 unique target genes, identified through literature and database searches to either be regulated by P53 or known to regulate P53 activity, were used for further analysis (Additional file 1, Table S1). Unpaired *t*-tests were used to identify P53 target transcripts with significantly altered expression (p < 0.05) between melanoma and normal cells; and between melanoma cells with mutant/null *P53 *compared to those with wild-type *P53*. One-way ANOVA with a post-hoc Tukey test was used to determine target genes regulated by P53 inhibition in multiple cell lines. To control for false positive results, Benjamini and Hochberg False Discovery Rate (FDR) of 5.0% was used for multiple testing. Genes that had more than 2 fold increase or decrease in expression, a p-value equal to or below 0.05 and an FDR that did not exceed 0.05 were considered to be differentially expressed between the two sample groups.

SOURCE [25] and PANTHER [26] were used to annotate the biological processes and pathways that differentially expressed genes were involved in. Differentially expressed gene lists were compared to the PANTHER reference list and to each other using the gene expression analysis tool. This tool uses the binomial test for each molecular function, biological process, or pathway term in PANTHER, to statistically (p < 0.05) determine over- or under-representation of PANTHER classification categories. A PANTHER category with a p-value equal to or below 0.05 was considered to significantly over- or under-represented. Supervised hierarchical cluster analysis was performed on genes that were found to be significantly different (> 2 fold, p < 0.05). Similarity in the expression patterns between genes was measured by Manhattan distance. The results of this microarray analysis were deposited in Gene Expression Omnibus (GEO) http://www.ncbi.nlm.nih.gov/geo/ with Accession No. GSE29377.

### Western blot analysis

Protein extraction, separation by SDS-PAGE and western blot analysis of cell lines to confirm inhibition of P53 expression was performed as described previously [16]. The mouse monoclonal antibodies used for the detection of P53 (BP53-12) and glyceraldehyde-3-phosphate dehydrogenase (GAPDH) were purchased from Upstate (NY, USA) and Ambion (TX, USA) respectively.

### Real-time PCR

Total RNA (500 ng) was reverse transcribed to generate cDNA using the High Capacity cDNA Reverse Transcription Kit (Applied Biosystems, VIC, Australia) according to the manufacturers' instructions. Real-time PCR analysis was performed in triplicate using TaqMan^® ^Universal PCR mix and TaqMan^® ^Gene Expression Assays (Applied Biosystems) according to the manufacturers' instructions, with results quantified on a 7500 real-time PCR system (Applied Biosystems). The expression of the following transcripts was analysed: *CDC25C *(Hs00156411_m1), *BIRC5 *(Hs00977611_g1), *CDKN2A *(Hs00923894_m1), *PLK2 *(Hs01573415_g1), *SESN1 *(Hs00205427_m1), *BRCA1 *(Hs01556191_m1) and *β-Actin *(4326315E). The relative expression of the gene of interest was normalised to *β-Actin *(DCt) and expressed as the fold change calculated using the 2^-ΔΔCt ^method [27].

### Cell proliferation assays

Colony formation and MTT assays were used to measure cellular proliferation and were performed as previously described [28].

## Results

### Transcript expression of P53 target genes in metastatic melanoma

**Figure 1:**
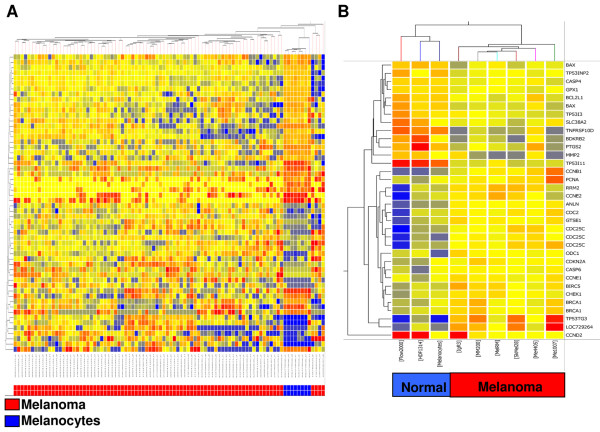
**The mRNA expression of P53 target genes is de-regulated in human melanoma**. Supervised hierarchical cluster analysis was performed on P53 target genes significantly altered between melanoma and melanocytes. Similarity in the mRNA expression patterns between genes and between samples was measured using Manhattan distance. Distances between clusters represent the average distances between genes and samples in the cluster. Genes are coloured according to their expression level, where up-regulated expression is represented by red, down-regulated expression is represented by blue, and equal expression is represented by yellow. **(A) **Analysis of 56 differentially expressed genes in 82 metastatic melanoma patients compared to 8 melanocyte cell lines. **(B) **Analysis of 34 differentially expressed genes in 6 melanoma cell lines (IgR3, Mel-RM, Me1007, MM200, Sk-Mel-28, Me4405) compared to normal cells (melanocytes, FLOW2000, HDF1314).

To determine genes involved in the P53 signalling pathway that were altered in metastatic melanoma, whole genome bead arrays were used to analyse gene expression patterns in 82 metastatic melanomas compared to 8 diploid melanocyte strains, which were used as a normal control. A literature and database search identified 290 probes present on the arrays, representing 247 unique transcripts and 181 unique target genes that were known to regulate or to be regulated by P53 (Additional file 1, Table S1), and these were further analysed between the two groups (metastatic melanoma versus melanocytes). Fifty-six of the transcripts (56/290, 19.3%) were identified as being differentially expressed between the metastatic melanomas and the melanocytes. Supervised hierarchical clustering of these genes clearly separated the melanocytes from the metastatic melanoma cases, suggesting that the mRNA expression of these P53 target genes can discriminate these two groups (Figure 1A).

Of the 56 significantly altered transcripts, 23 showed increased expression and 33 showed decreased expression in metastatic melanomas when compared to melanocytes (Table 1). Apoptosis and cell cycle were the main P53-regulated biological processes altered in metastatic melanoma, representing 23.2% (13/56) and 28.6% (16/56) of the gene set respectively (Table 1). The majority of transcripts (9/13, 69.2%) involved in apoptosis regulation were significantly decreased in metastatic melanoma when compared to melanocytes and included the Bcl-2 family members *BAX *and *Bcl-xL (BCL2L1*); *Caspases 6, 7 *and *8*; and the tumour necrosis factor receptor superfamily member 10D (*TRAIL-R4, DcR2*) (Table 1). The mRNA expression of several genes involved in cell cycle control and/or proliferation, including the *Cyclins B3, E1, G1 *and *G2; PCNA *and *RB1 *were also decreased in metastatic melanoma (Table 1). Decreased expression of Cyclin D1 and RB1 proteins has previously been reported in melanoma [5]. Several genes involved in immunity and defense were highly over-expressed in metastatic melanomas (Table 1). Of these, the mRNA expression of *THBS1 *and *THBS2 *has been inversely associated with melanoma growth and progression [29,30], while *CX3CL1 *inhibition has been shown to reduce melanoma growth and angiogenesis in mice [31].

**Table 1:** P53 targets differentially expressed in melanoma metastases

No.	Accession No.	Gene Symbol	Gene Name	Fold change	p-value
**Apoptosis**					
**1**	NM_001040619.1	ATF3	Activating transcription factor 3, transcript variant 4	4.62	0.0042
**2**	NM_003879.3	CFLAR/FLIP	CASP8 and FADD-like apoptosis regulator	3.10	0.0063
**3**	NM_004324.3	BAX	BCL2-associated X protein, transcript variant beta	-3.92	0.0127
**4**	NM_138578.1	BCL2L1	BCL2-like 1 (Bcl-xL), transcript variant 1	-5.77	0.0144
**5**	NM_001226.3	CASP6	Caspase 6, transcript variant alpha	-13.07	4.98E-05
**6**	NM_033340.2	CASP7	Caspase 7, transcript variant beta	-3.69	0.0465
**7**	NM_001080125.1	CASP8	Caspase 8, transcript variant G	-11.38	2.28E-05
**8**	NM_033356.3	CASP8	Caspase 8, transcript variant C	-2.96	0.0273
**9**	NM_001007277.1	EI24	Etoposide induced 2.4 mRNA, transcript variant 2	-2.81	0.0245
**10**	NM_021127.1	PMAIP1	Phorbol-12-myristate-13-acetate-induced protein 1 (Noxa)	12.18	0.0063
**11**	NM_000314.4	PTEN	Phosphatase and tensin homolog	-3.29	0.0062
**12**	NM_003840.3	TNFRSF10D	Tumor necrosis factor receptor superfamily, member 10d	-29.89	8.37E-07
**13**	NM_004881.2	TP53I3	Tumor protein p53 inducible protein 3, transcript variant 1	-4.50	0.0097
**Cell cycle, proliferation and differentiation**					
**14**	NM_033031.2	CCNB3	Cyclin B3, transcript variant 3	-8.38	1.47E-04
**15**	NM_033031.2	CCNB3	Cyclin B3, transcript variant 3	-10.79	1.18E-04
**16**	NM_001759.2	CCND2	Cyclin D2	76.10	1.73E-07
**17**	NM_001238.1	CCNE1	Cyclin E1, transcript variant 1	-7.61	2.07E-10
**18**	NM_057749.1	CCNE2	Cyclin E2	3.54	0.0476
**19**	NM_199246.1	CCNG1	Cyclin G1, transcript variant 2	-5.75	0.0146
**20**	NM_004354.1	CCNG2	Cyclin G2	-10.66	2.10E-04
**21**	NM_001798.2	CDK2	Cyclin-dependent kinase 2, transcript variant 1	-4.09	0.0476
**22**	NM_001798.2	CDK2	Cyclin-dependent kinase 2, transcript variant 1	-17.99	0.0017
**23**	NM_058197.3	CDKN2A	Cyclin-dependent kinase inhibitor 2A, transcript variant 3	4.97	0.0202
**24**	NM_058195.2	CDKN2A	Cyclin-dependent kinase inhibitor 2A, transcript variant 4	-7.09	0.0351
**25**	NM_015675.2	GADD45B	Growth arrest and DNA-damage-inducible, beta	7.41	0.0020
**26**	NM_006705.2	GADD45G	Growth arrest and DNA-damage-inducible, gamma	11.13	0.0154
**27**	NM_002592.2	PCNA	Proliferating cell nuclear antigen, transcript variant 1	-7.25	4.32E-04
**28**	NM_182649.1	PCNA	Proliferating cell nuclear antigen, transcript variant 2	-4.53	0.0219
**29**	NM_000321.2	RB1	Retinoblastoma 1	-5.85	0.0097
**DNA repair**					
**30**	NM_138292.3	ATM	Ataxia telangiectasia mutated, transcript variant 2	4.49	0.0328
**31**	NM_007306.2	BRCA1	Breast cancer 1, early onset, transcript variant BRCA1-exon4	-3.56	0.0371
**Immunity and defense**					
**32**	NM_001024844.1	CD82	CD82 molecule, transcript variant 2	-4.80	0.0127
**33**	NM_002996.3	CX3CL1	Chemokine (C-X3-C motif) ligand 1	9.44	2.67E-05
**34**	NM_153201.1	HSPA8	Heat shock 70kDa protein 8, transcript variant 2	-3.47	0.0350
**35**	NM_006597.3	HSPA8	Heat shock 70kDa protein 8, transcript variant 1	-3.31	0.0100
**36**	NM_001098631.1	IRF5	Interferon regulatory factor 5, transcript variant 7	13.39	3.78E-04
**37**	NM_182826.1	SCARA3	Scavenger receptor class A, member 3, transcript variant 2	6.60	0.0139
**38**	NM_003246.2	THBS1	Thrombospondin 1	81.54	1.31E-08
**39**	NM_003247.2	THBS2	Thrombospondin 2	4.16	0.0408
**Metabolism**					
**40**	NM_020128.1	MDM1	Mdm4, transformed 3T3 cell double minute 1, transcript variant 2	-3.98	0.0146
**41**	NM_020128.1	MDM1	Mdm4, transformed 3T3 cell double minute 1, transcript variant 2	7.64	0.0023
**42**	NM_004530.2	MMP2	Matrix metallopeptidase 2	-12.58	0.0017
**43**	NM_000603.3	NOS3	Nitric oxide synthase 3	11.77	4.62E-05
**44**	NM_033239.2	PML	Promyelocytic leukemia, transcript variant 9	-6.24	2.81E-04
**45**	NM_001034.1	RRM2	Ribonucleotide reductase M2 polypeptide	-3.38	0.0476
**46**	NM_005063.4	SCD	Stearoyl-CoA desaturase	-4.04	0.0039
**Transcription regulation**					
**47**	NM_006210.1	PEG3	Paternally expressed 3	42.85	2.28E-05
**48**	NM_003068.3	SNAI2/SLUG	Snail homolog 2	-27.69	9.45E-07
**49**	NM_004295.3	TRAF4	TNF receptor-associated factor 4	3.75	0.0033
**50**	NM_152240.1	ZMAT3/WIG1	Zinc finger, matrin type 3, transcript variant 2	-6.97	0.0024
**Signal transduction**					
**51**	NM_014376.2	CYFIP2	Cytoplasmic FMR1 interacting protein 2, transcript variant 3	17.86	1.20E-08
**52**	NM_004431.2	EPHA2	EPH receptor A2	5.42	0.0031
**53**	NM_001005914.1	SEMA3B	Semaphorin 3B, transcript variant 2	156.75	1.59E-08
**Unknown function**					
**54**	NM_012242.2	DKK1	Dickkopf homolog 1	-17.41	0.0097
**55**	NM_182915.2	STEAP3	STEAP family member 3, transcript variant 1	8.22	0.0024
**56**	NM_005802.2	TOPORS	Topoisomerase I binding, arginine/serine-rich	-4.13	0.0350

Fold change in mRNA expression of P53 target genes found to be significantly different in extracts from 82 metastatic melanomas compared to extracts from 8 melanocyte cell lines (> 2-fold difference, p < 0.05 and a false discovery rate (FDR) = 5.0%).

### Transcript expression of P53 target genes in melanoma cell lines

To determine whether altered regulation of P53 target genes could be recapitulated *in vitro*, the mRNA expression of the 290 probes (Additional file 1, Table S1) were examined in six melanoma cell lines, including four with wild-type *P53 *(IgR3, Mel-RM, MM200, Me1007), one with no *P53 *expression (Me4405) and one with mutant *P53 *(Sk-Mel-28, G454A) [16]. The mRNA expression of these genes in melanoma cells was compared to a melanocyte cell line and two fibroblast strains (HDF1314, FLOW2000), which served as normal controls. Thirty-four transcripts (34/290, 11.72%) were significantly altered in melanoma cells when compared to normal cells (Table 2) and could clearly distinguish normal cells from the melanoma cell lines in hierarchical cluster analysis (Figure 1B).

**Table 2:** P53 targets differentially expressed in melanoma cells

No.	Accession No.	Gene Symbol	Gene Name	Fold change	p-value
**Apoptosis**					
*Induction of apoptosis*					
**1**	NM_138765.2	BAX	BCL2-associated X protein, transcript variant sigma	-4.19	0.0062
**2**	NM_004324.3	BAX**^1^**	BCL2-associated X protein, transcript variant beta	-4.67	0.0059
**3**	NM_001225.3	CASP4	Caspase 4, transcript variant alpha	-2.32	0.0027
**4**	NM_001226.3	CASP6**^1^**	Caspase 6, transcript variant alpha	3.17	0.0401
**5**	NM_021202.1	TP53INP2	Tumor protein p53 inducible nuclear protein 2	-3.02	0.0034
**6**	NM_147184.1	TP53I3/PIG3	Tumor protein p53 inducible protein 3, transcript variant 2	-3.67	0.0031
*Inhibition of apoptosis*					
**7**	NM_138578.1	BCL2L1**^1^**	B-cell CLL/lymphoma 2-like 1, transcript variant 1	-2.95	0.0124
**8**	NM_003840.3	TNFRSF10D**^1^**	Tumor necrosis factor receptor superfamily, member 10d	-33.80	0.0005
**Cell cycle, proliferation and differentiation**					
**9**	NM_001012271.1	BIRC5	Baculoviral IAP repeat-containing 5 (Survivin), transcript variant 3	4.60	0.0024
		*BIRC5*		*12.61*	*1.8E-05*
**10**	NM_031966.2	CCNB1	Cyclin B1	18.17	0.0005
**11**	NM_001759.2	CCND2**^1^**	Cyclin D2	-75.73	0.0427
**12**	NM_001238.1	CCNE1**^1^**	Cyclin E1, transcript variant 1	2.34	0.0310
**13**	NM_057735.1	CCNE2	Cyclin E2, transcript variant 2	9.33	0.0198
**14**	NM_001786.2	CDC2/CDK1	Cell division cycle 2, transcript variant 1	6.32	0.0127
**15**	NM_001790.3	CDC25C	Cell division cycle 25 homolog C, transcript variant 1	17.05	0.0020
**16**	NM_001790.3	CDC25C	Cell division cycle 25 homolog C, transcript variant 1	15.26	0.0001
**17**	NM_022809.2	CDC25C	Cell division cycle 25 homolog C, transcript variant 2	13.39	0.0011
		*CDC25C*		*18.21*	*0.0004*
**18**	NM_058197.3	CDKN2A**^1^**	Cyclin-dependent kinase inhibitor 2A, transcript variant 3	3.14	0.0472
		*CDKN2A*		*1.56*	*0.4706*
**19**	NM_001274.3	CHEK1	CHK1 checkpoint homolog	3.14	0.0105
**20**	NM_016426.4	GTSE1	G-2 and S-phase expressed 1	6.69	0.0014
**21**	NM_182649.1	PCNA**^1^**	Proliferating cell nuclear antigen, transcript variant 2	4.66	0.0397
**22**	NM_006034.2	TP53I11	Tumor protein p53 inducible protein 11	-54.31	2.31E-06
**DNA repair**					
**23**	NM_007304.2	BRCA1	Breast cancer 1, transcript variant BRCA1-delta11b	3.70	0.0092
**24**	NM_007299.2	BRCA1	Breast cancer 1, transcript variant BRCA1-delta14-17	3.06	0.0068
		*BRCA1*		*5.47*	*0.0037*
**Immunity and defense**					
**25**	NM_000963.1	PTGS2	Prostaglandin-endoperoxide synthase 2	-15.59	0.0074
**Metabolism**					
**26**	NM_201397.1	GPX1	Glutathione peroxidase 1, transcript variant 2	-2.16	0.0011
**27**	NM_004530.2	MMP2	Metallopeptidase 2	-6.61	0.0296
**28**	NM_001034.1	RRM2**^1^**	Ribonucleotide reductase M2 polypeptide	10.48	0.0068
**Signal Transduction**					
**29**	NM_000623.2	BDKRB2	Bradykinin receptor B2	-16.54	0.0040
**Transport**					
**30**	NM_002539.1	ODC1	Ornithine decarboxylase 1	4.12	0.0099
**31**	NM_018976.3	SLC38A2	Solute carrier family 38, member 2	-5.03	0.0074
**Biological function unclassified**					
**32**	NM_018685.2	ANLN	Anillin	8.88	4.17E-06
**33**	XM_001133677.1	LOC729264	PREDICTED: Similar to TP53TG3 protein, transcript variant 2	25.41	0.0068
**34**	NM_016212.2	TP53TG3	Tumor protein p53 target gene 3	102.59	0.0005

Fold change in mRNA expression of 34 P53 target genes found to be significantly different in melanoma cell lines (IgR3, Mel-RM, MM200, Me1007, Me4405, Sk-Mel-28) compared to normal cell lines (melanocytes, FLOW2000, HDF1314) (> 2-fold difference, p ≤ 0.05 and FDR = 5.0%). Real-time qRT-PCR verification of selected genes is shown in italics. **^1^**Genes found to be differentially expressed between metastatic melanoma patients and melanocytes.

Of these transcripts, 9/34 (26.5%) were identified as being differentially expressed in metastatic melanoma patients when compared to melanocytes (Table 2). Although some of these genes (e.g. cell cycle genes *CCND2, CCNE1 *and *PCNA*) had fold changes that were in a different direction when compared to metastatic melanoma patients (Tables 1 and 2), this is likely to be due to the active growth of these cells in culture. The majority of transcripts found to be significantly different in melanoma when compared to normal cells were involved in apoptosis (8/34, 23.5%) or cellular proliferation and/or differentiation (16/34, 47.1%) (Table 2). Overall, the mRNA expression of P53 target genes involved in apoptosis was significantly decreased in melanoma, while the mRNA expression of P53 target genes involved in cell cycle regulation was significantly increased (Table 2). These results suggest that the mRNA expression profiles of P53-regulated target genes and the P53-regulated biological processes that are altered as a result of their changed expression are similar between metastatic melanoma patients and melanoma cell lines, further confirming the disruption of P53-regulated apoptotic and cell cycle pathways in melanoma.

The expression of these transcripts in cell lines with wild-type *P53 *(IgR3, Me1007, Mel-RM, MM200) was similar in the two cell lines which had null/mutant *P53 *expression, Me4405 and Sk-Mel-28, suggesting that overall, their expression was not related to *P53 *status (Figure 1B). In confirmation, the mRNA expression of very few P53 target genes was found to be significantly different between cell lines with wild-type *P53 *when compared to cell lines with null/mutant *P53 *expression (Table 3). The mRNA expression of *Stromal antigen 1 (STAG1) *and *Survivin (BIRC5) *was significantly higher in cell lines with wild-type *P53 *compared to those with null/mutant *P53*; whereas *Caspase 8 (CASP8), Snail homolog 2 (SLUG), Cell division cycle 25C (CDC25C), CD82 (KAI1) *and *P14ARF (CDKN2A) *were expressed at significantly lower levels (Table 3). Thus, these results suggest that the expression or mutation status of *P53 *in melanoma has little impact on the expression profile of P53 target genes.

**Table 3:** P53 target genes differentially expressed in melanoma cells with wild-type or mutant P53

Accession No.	Gene Symbol	Gene Name	Fold change (WT vs MT)	p-value
NM_001012271.1	BIRC5	Baculoviral IAP repeat-containing 5 (Survivin), transcript variant 3	2.81	0.03297
	*BIRC5*		*2.42*	*0.01001*
NM_001080125.1	CASP8	Caspase 8, transcript variant G	-5.88	0.02569
NM_001024844.1	CD82	CD82 molecule, transcript variant 2	-19.52	0.03576
NM_001790.3	CDC25C	Cell division cycle 25 homolog C, transcript variant 1	-2.57	0.00007
NM_058195.2	CDKN2A	Cyclin-dependent kinase inhibitor 2A, transcript variant 4	-88.58	0.0159
	*CDKN2A*		*-34.08*	*2.2E-05*
NM_003068.3	SNAI2/SLUG	Snail homolog 2	-8.92	0.00005
NM_005862.2	STAG1	Stromal antigen 1	2.33	0.04536

Fold change in mRNA expression of p53 target genes found to be significantly different in extracts from melanoma cell lines expressing wild-type p53 (IgR3, Mel-RM, MM200, Me1007) compared to melanoma cell lines with null/mutant p53 (Sk-Mel-28, Me4405) (> 2-fold difference, p ≤ 0.05 and FDR = 5.0%). Real-time qRT-PCR verification of selected genes is shown in italics.

### Inhibition of P53 has limited effect on the mRNA expression of known P53 target genes in melanoma

To formally test the role of P53 in the regulation of these P53-regulated transcripts, melanocytes and melanoma cell lines (Mel-RM and IgR3) were generated in which the expression of the P53 protein was stably inhibited using shRNA. These were compared to cells which had been stably transduced with a non-specific control shRNA. Of the 290 transcripts analysed (Additional file 1, Table S1), inhibition of P53 expression resulted in differential regulation of 19 (6.6%) transcripts in melanocytes. In melanoma cells, approximately half the number of transcripts were shown to be significantly regulated by P53 (7 (2.4%) in IgR3, 11 (3.8%) in Mel-RM), further suggesting the lack of P53 regulation of common target genes in melanoma (Table 4).

**Table 4:** P53 target genes regulated by P53 knockdown

Accession No.	Gene Symbol	Gene Name	Melan.	IgR3	Mel-RM
***Genes regulated similarly by p53 KO in melanocytes and melanoma cells***					
*Cell cycle, proliferation or differentiation*					
NM_000389.2	CDKN1A	Cyclin-dependent kinase inhibitor 1A, transcript variant 1	**2.68**	-	**7.12**
NM_004864.1	GDF15	Growth differentiation factor 15	**3.42**	-	**6.11**
*Transcription regulation/signal transduction*					
NM_014376.2	CYFIP2	Cytoplasmic FMR1 interacting protein 2, transcript variant 3	**5.11**	-	**8.88**
***Genes regulated by p53 KO in melanoma cells but not in melanocytes***					
*Apoptosis*					
NM_138578.1	BCL2L1**^1^**	BCL2-like 1 (Bcl-xL), transcript variant 1	-	**-2.06**	**2.01**
NM_033294.2	CASP1	Caspase 1, transcript variant delta	-	-	**-4.20**
NM_016479.3	SHISA5	Shisa homolog 5	-	**-2.30**	-
NM_019058.2	DDIT4	DNA-damage-inducible transcript 4	-	**-2.56**	-
*Cell cycle, proliferation or differentiation*					
NM_078467.1	CDKN1A	Cyclin-dependent kinase inhibitor 1A, transcript variant 2	-	-	**7.56**
NM_000548.3	TSC2	Tuberous sclerosis 2, transcript variant 1	-	-	**3.95**
*Immunity and defense*					
NM_003897.3	IER3	Immediate early response 3	-	-	**2.59**
*Metabolism*					
NM_000603.3	NOS3	Nitric oxide synthase 3	-2.72	**10.65**	-
*Transcription regulation/signal transduction*					
NM_001037333.1	CYFIP2	Cytoplasmic FMR1 interacting protein 2, transcript variant 1	-	-	**14.63**
NM_152546.1	SRFBP1	Serum response factor binding protein 1	-	-	**-2.12**
NM_004559.3	YBX1	Y box binding protein 1	-	**-2.75**	-
*Transport*					
NM_000593.5	TAP1	Transporter 1	-	**2.07**	-
*Unknown function*					
NM_182915.2	STEAP3	STEAP family member 3, transcript variant 1	-	**4.64**	**-2.94**
***Genes regulated by p53 KO in melanocytes but not in melanoma***					
*Apoptosis*					
NM_001040619.1	ATF3	Activating transcription factor 3, transcript variant 4	**-5.30**	-	-
NM_001008925.1	RCHY1	Ring finger and CHY zinc finger domain containing 1, transcript variant 2	**-12.23**	-	-
NM_005427.1	TP73	Tumor protein p73	**10.54**	-	2.84
*Cell cycle, proliferation or differentiation*					
NM_001012271.1	BIRC5**^1^**	Baculoviral IAP repeat-containing 5, transcript variant 3	**-18.95**	-	-
NM_004701.2	CCNB2	Cyclin B2	**-3.17**	-	-
NM_057735.1	CCNE2**^1^**	Cyclin E2, transcript variant 2	**-5.73**	-	-
NM_001786.2	CDC2**^1^**	Cell division cycle 2, transcript variant 1	**-8.42**	-	-
NM_022809.2	CDC25C**^1^**	Cell division cycle 25 homolog C, transcript variant 2	**-9.40**	-	-
NM_001259.5	CDK6	Cyclin-dependent kinase 6	**2.15**	-	-
NM_058195.2	CDKN2A**^1^**	Cyclin-dependent kinase inhibitor 2A, transcript variant 4	**-12.78**	-	-
NM_006622.2	PLK2	Polo-like kinase 2	**18.00**	-8.19	-
NM_014454.1	SESN1	Sestrin 1	**2.69**	-	-
*Immunity and defense*					
NM_003246.2	THBS1	Thrombospondin 1	**-18.25**	-	-
NM_003247.2	THBS2	Thrombospondin 2	**2.21**	-	-
NM_033550.3	TP53RK	TP53 regulating kinase	**-2.08**	-	-
*Transcription regulation/signal transduction*					
NM_000376.2	VDR	Vitamin D receptor, transcript variant 1	**-20.88**	-	-

Fold change in mRNA expression of P53 targets genes regulated in melanocyte (Melan.), IgR3 or Mel-RM cell lines in which P53 expression had been inhibited compared to cells expressing normal levels of P53 (control shRNA versus *P53 *shRNA). Genes that were significantly different (> 2-fold change, p ≤ 0.05 and FDR = 5.0%) in comparisons of control shRNA with *P53 *shRNA are shown in bold, while genes which were not regulated (< 2-fold change) are represented by a hyphen (-). **^1^**Genes found to be differentially expressed between melanoma cells and normal cells.

**Figure 2:**
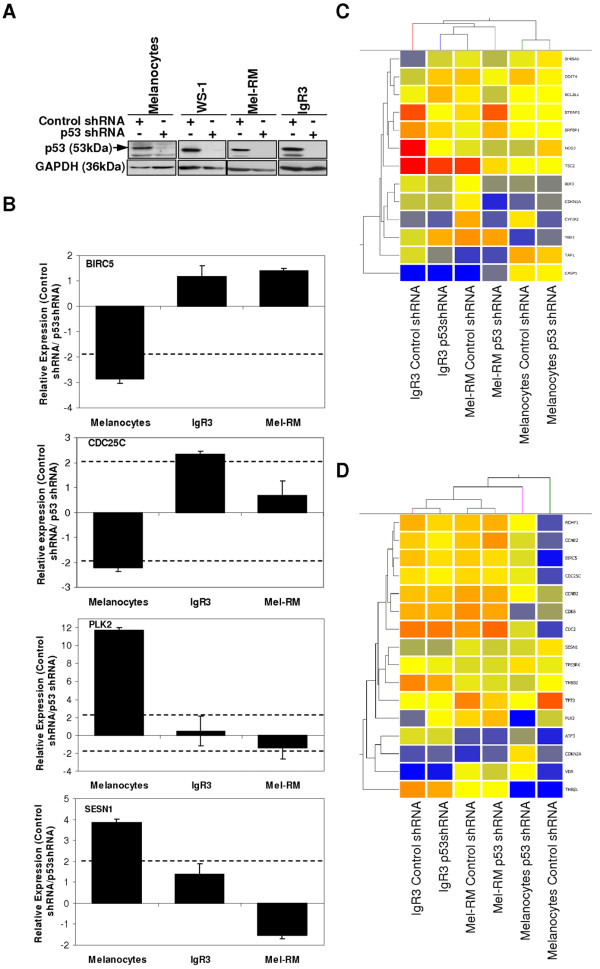
**Inhibition of P53 expression by shRNA alters regulation of P53 target genes**. **(A) **Protein (25 μg) from melanocytes, WS-1, Mel-RM and IgR3 cells that had been stably transduced with *P53 *shRNA or control shRNA was analysed for the expression of P53 by western blotting. The expression of GAPDH was determined to ensure equal loading. Arrowhead indicates expected molecular weight. **(B) **Relative quantification of *BIRC5, CDC25C, PLK2 and SESN1 *mRNA by real-time RT-PCR in melanocytes, Mel-RM and IgR3 cells that had been stably transduced with *P53 *shRNA or control shRNA. Results are shown as the relative normalised expression (target/*β-Actin*) of the target gene in cells transduced with control shRNA compared to cells transduced with *P53 *shRNA (2^-ΔΔCt^). Values represent the mean ± SE. **(C) **Supervised hierarchical cluster analysis of 13 genes that were regulated by P53 in melanoma cells only and not in melanocytes. Genes are coloured according to their expression level, where up-regulated expression is represented by red, down-regulated expression is represented by blue, and equal expression is represented by yellow. **(D) **Supervised hierarchical cluster analysis of 16 genes that were regulated by P53 in melanocytes only and not in melanoma cells. Genes are coloured according to their expression level, where up-regulated expression is represented by red, down-regulated expression is represented by blue, and equal expression is represented by yellow.

Only 3 genes, *P21 (CDKN1A), Growth differentiation factor 15 (GDF15) *and *Cytoplasmic FMR1 interacting protein 2 (CYFIP2) *were commonly regulated in melanocytes and melanoma cells alike and the direction of their regulation (i.e. increased transcript expression of *P21 *in control shRNA transduced cells) was consistent with their expected regulation by P53 (Table 4). Of note, a high proportion of genes (16/19) that were regulated by P53 in melanocytes were not regulated in melanoma cells. In particular, the mRNA expression of P53 target genes involved in cell cycle regulation were predominantly altered by P53 inhibition in melanocytes and these genes showed a complete lack of regulation by P53 in melanoma cell lines (Table 4). Many of the P53 target genes involved in cell cycle regulation (5/9 genes, Table 4) that were altered by P53 inhibition in melanocytes but not in melanoma, were over-expressed in melanoma when compared to normal cells (Table 2). Notably, the mRNA expression of *CDKN2A *and *BIRC5 *(shown to be altered in *P53 *mutant melanoma cells) was significantly lower in melanocytes expressing control shRNA, suggesting that the inhibitory effect of P53 expression on these genes was relieved in cells expressing *P53 *shRNA (Table 4). However, P53 inhibition had no effect on *CDKN2A *or *BIRC5 *transcript expression in melanoma cells (Table 4), confirming de-regulated signalling by P53 in these cells. The lack of P53-dependent regulation of its target genes in melanoma was not due to a failure to inhibit this protein, given that P53 expression was shown to be almost completely abolished in cells transduced with *P53 *shRNA (Figure 2A). Moreover, the altered P53-dependent transcriptional regulation of 4 genes *(BIRC5, CDC25C, PLK2 *and *SESN1) *in melanoma compared to melanocytes was confirmed by real-time PCR (Figure 2B). In addition, we have previously shown that endogenous and over-expressed P53 can regulate the transcription of the *P21 *and *PUMA *promoters in luciferase assays [16] and in this study, both the basal and cisplatin-induced protein expression of P21 was abolished in IgR3 and Mel-RM cells in which P53 had been inhibited (Additional file 2, Figure S1), demonstrating that P53 in these cells is transcriptionally competent. Taken together, these results suggest that the constitutive transcriptional regulation of known P53 target genes involved in the cell cycle is considerably dampened in melanoma.

### Inhibition of P53 in melanocytes results in an altered P53 target gene mRNA expression profile that is similar to that observed in melanoma cells

Hierarchical cluster analysis of the 13 genes that were only regulated by P53 in melanoma cells and not in melanocytes (Table 4) could not distinguish melanoma cells that had been transduced with *P53 *shRNA from melanoma cells that had been transduced with control shRNA (Figure 2C). However, hierarchical cluster analysis of the 16 genes that were significantly regulated by P53 only in melanocytes and not in melanoma cells (Table 4), clearly separated the cell lines into two distinct groups (Figure 2D). Melanocytes that had been transduced with control shRNA formed one branch of the dendrogram, while melanocytes that had been transduced with *P53 *shRNA formed another branch that was more closely related to all of the melanoma cell lines (regardless of P53 expression) than it was to melanocytes transduced with control shRNA (Figure 2D). These results suggest that transcriptional control of key P53 target genes (mostly involved in cell cycle) by P53 in melanocytes is necessary for normal function and that disrupted transcriptional regulation of these target genes (by inhibition of P53) can induce gene expression profiles that are similar to that observed in melanoma cells.

### The ability of P53 to regulate genes involved in the cell cycle is significantly reduced in melanoma cells

**Figure 3:**
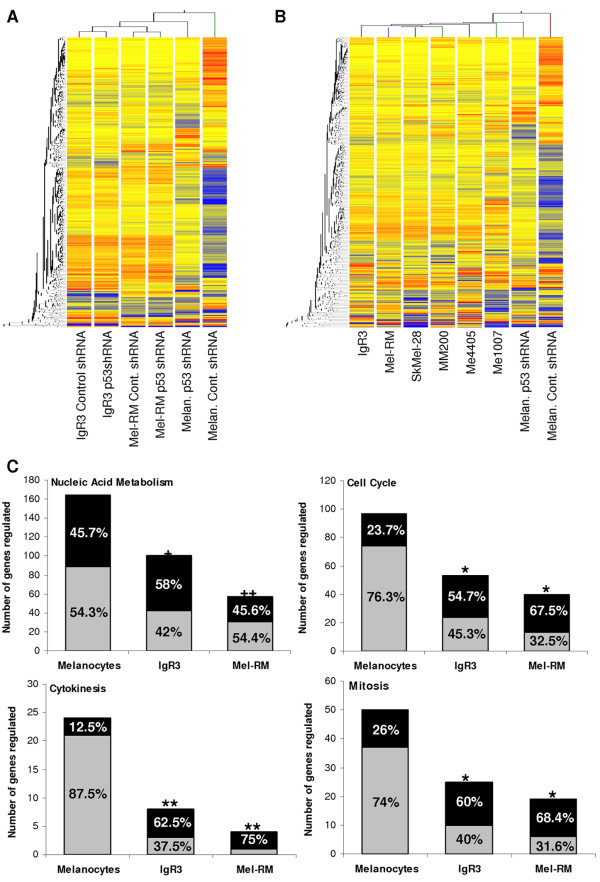
**Ability of P53 to regulate genes involved in cell cycle is significantly reduced in melanoma**. **(A) **Supervised hierarchical cluster analysis of 728 genes that were significantly regulated by P53 in melanocytes. The relative mRNA expression of these genes in melanocytes, Mel-RM and IgR3 cells that had been stably transduced with either *P53 *shRNA or control shRNA is shown. **(B) **Supervised hierarchical cluster analysis of 728 genes that were significantly regulated by P53 in melanocytes. The relative mRNA expression of these genes in melanocytes that had been stably transduced with either *P53 *shRNA or control shRNA compared to IgR3, Mel-RM, SkMel-28, MM200, Me4405, and Me1007 melanoma cell lines is shown. Genes are coloured according to their expression level, where up-regulated expression is represented by red, down-regulated expression is represented by blue, and equal expression is represented by yellow. **(C) **The number of genes regulated by P53 (control shRNA versus *P53 *shRNA) in melanocytes, IgR3 and Mel-RM cell lines in the biological process categories: nucleic acid metabolism, cell cycle, cytokinesis and mitosis as defined by PANTHER [26]. Up-regulated genes are shown in black while down-regulated genes are shown in grey. The number of genes regulated are also depicted as percentages of the total gene list on the bar graph for each of the cell lines. The significance of the regulation of these biological processes by P53 in each of the melanoma cell lines (Mel-RM and IgR3) compared to melanocytes was determined using the gene expression tool in PANTHER (^+^p < 0.0005, ^++^p = 0.000003, *p < 0.05, **p < 0.005).

Given that P53 in melanoma cells failed to regulate typical P53 target genes when compared to melanocytes, we next determined the effect of P53 knockdown on whole genome gene expression profiles. Of the 24,526 transcripts analysed, inhibition of P53 expression resulted in differential regulation of 728 (2.97%) transcripts in melanocytes. In melanoma cells, fewer transcripts were shown to be significantly regulated by P53; 591 transcripts (2.41%) in IgR3 and 398 transcripts (1.62%) in Mel-RM. Hierarchical clustering of the 728 transcripts regulated by P53 in melanocytes showed that very few of these target genes were regulated in the IgR3 and Mel-RM melanoma cell lines by P53 (Figure 3A). Furthermore, following P53 knockdown the expression of these transcripts in melanocytes was highly similar to the expression of these transcripts in melanoma cells, where two distinct groupings were seen in hierarchical clustering. Melanocytes that had been transduced with control shRNA formed one group, while melanoma cell lines and melanocytes with inhibited P53 expression formed another group (Figure 3A and 3B). These results further confirmed that disrupted regulation of P53 signalling in melanocytes can induce gene expression profiles that are similar to those observed in melanoma cells.

To determine the biological processes that were significantly altered by P53 knockdown, the gene ontologies of the significantly regulated genes (control shRNA versus P53 shRNA) in each of the cell lines were analysed in PANTHER [26]. Mitosis, cellular processes, cell cycle, cytokinesis and nucleic acid metabolism were the 5 most significantly up-regulated processes in melanocytes (Table 5). Cellular processes were also significantly up-regulated in both melanoma cell lines, while mitosis and cell cycle were significantly up-regulated in Mel-RM and IgR3 cells respectively (Table 5). These results are concordant with a recent study conducted by Terzian and colleagues who found that activation of P53 by Nutlin in primary melanocytes repressed the expression of genes involved in cell cycle progression, DNA replication and chromosomal maintenance [32]. We next determined whether the biological processes regulated by P53 knockdown in melanoma cell lines were different to those that were regulated by P53 knockdown in melanocytes. Genes involved in mitosis, cell cycle, cytokinesis and nucleic acid metabolism were significantly under-represented in both Mel-RM and IgR3 cell lines when compared to melanocytes (Table 5 and Figure 3C). In addition, while the majority of transcripts involved in mitosis, cell cycle and cytokinesis were down-regulated in control shRNA melanocytes when compared to P53 knockdown melanocytes, the majority of genes regulated by P53 in these categories were up-regulated in melanoma cells (compare grey and black proportion of the bar graph in Figure 3C). This suggests that both the number of genes regulated by P53 and the direction of their regulation is significantly altered in melanoma cells when compared to melanocytes.

**Table 5:** Gene ontologies regulated by P53 knockdown

	Biological Process	% of genes regulated by p53 in Melan. (from 673 unique genes)	Over/under	p-value	% of genes regulated by p53 in IgR3 (from 552 unique genes)	Over/under	p-value	% of genes regulated by p53 in Mel-RM (from 380 unique genes)	Over/under	p-value
**1**	**Mitosis**	7.28	+	1.36E-07	**4.53**	**+**	**5.32E-02**	**5.00**	**+**	**3.80E-02**
**2**	Cellular process	40.86	+	1.55E-07	40.40	+	5.36E-06	**41.32**	**+**	**3.07E-05**
**3**	**Cell cycle**	14.71	+	3.28E-06	**11.78**	**+**	**2.68E-02**	**11.58**	**+**	**7.22E-02**
**4**	**Cytokinesis**	3.42	+	1.05E-05	**1.45**	**+**	**3.41E-01**	**1.05**	**-**	**5.23E-01**
**5**	**Nucleobase, nucleoside, nucleotide and nucleic acid metabolic process**	25.85	+	1.50E-05	**19.57**	**+**	**4.33E-01**	**16.05**	**-**	**6.48E-02**
**6**	Metabolic process	48.89	+	6.80E-05	47.83	+	1.60E-03	**43.68**	**+**	**2.10E-01**
**7**	Primary metabolic process	47.25	+	6.93E-05	45.83	+	2.78E-03	**41.84**	**+**	**2.38E-01**
**8**	Chromosome segregation	2.82	+	1.09E-04	**0.91**	**-**	**4.93E-01**	1.84	+	1.03E-01
**9**	Vesicle-mediated transport	9.06	+	5.16E-04	9.42	+	5.34E-04	8.95	+	9.33E-03
**10**	Anatomical structure morphogenesis	8.62	+	1.03E-03	8.70	+	2.18E-03	8.95	+	5.71E-03
**11**	Cellular component morphogenesis	8.62	+	1.03E-03	8.70	+	2.18E-03	8.95	+	5.71E-03
**12**	Ectoderm development	10.40	+	1.26E-03	10.14	+	5.92E-03	11.05	+	3.70E-03
**13**	Localization	1.63	+	1.27E-03	1.81	+	9.74E-04	0.79	+	3.35E-01
**14**	Nervous system development	9.36	+	1.38E-03	8.33	+	3.56E-02	10.00	+	3.79E-03
**15**	Cellular component organization	10.40	+	1.70E-03	11.05	+	7.71E-04	10.26	+	1.87E-02
**16**	Cell-cell signaling	9.66	+	2.12E-03	**11.96**	+	**4.33E-06**	7.63	+	2.57E-01
**17**	Lipid metabolic process	8.32	+	2.56E-03	9.06	+	7.31E-04	7.89	+	4.00E-02
**18**	Defense response to bacterium	1.19	+	3.85E-03	1.27	+	4.74E-03	0.53	+	4.06E-01
**19**	System process	14.41	+	5.19E-03	14.86	+	4.42E-03	13.68	+	6.96E-02
**20**	Meiosis	2.08	+	6.73E-03	1.27	+	2.86E-01	2.11	+	3.29E-02

Analysis of the top 20 biological processes regulated by P53 knockdown (control shRNA versus *P53 *shRNA) in melanocytes (Melan.) compared to IgR3 and Mel-RM cell lines using the PANTHER database and gene expression analysis tool [26]. The data are represented as the percentage of genes regulated in each category from the entire gene list (> 2-fold change, p ≤ 0.05, FDR = 5.0% in comparisons of control shRNA with *P53 *shRNA) for each cell line. P-values for biological processes that were significantly over or under represented (+/-) in comparisons of the gene lists between P53-regulated genes (from Melanocytes, IgR3 or Mel-RM) and the human reference list are shown in the table, while biological processes that were significantly different (p < 0.05) between melanocytes and either of the melanoma cell lines are highlighted in bold.

Taken together, these results suggest that P53 knockdown in melanocytes induced changes in gene expression patterns that were similar to the gene expression patterns in melanoma cells. Furthermore, many of the target genes that were regulated by P53 in melanocytes were unaffected in melanoma cells and in particular, the ability of P53 to regulate genes involved in cell cycle functions was significantly reduced in melanoma cells when compared to melanocytes.

### Inhibition of P53 increases proliferation in normal cells, but reduces proliferation in melanoma

**Figure 4:**
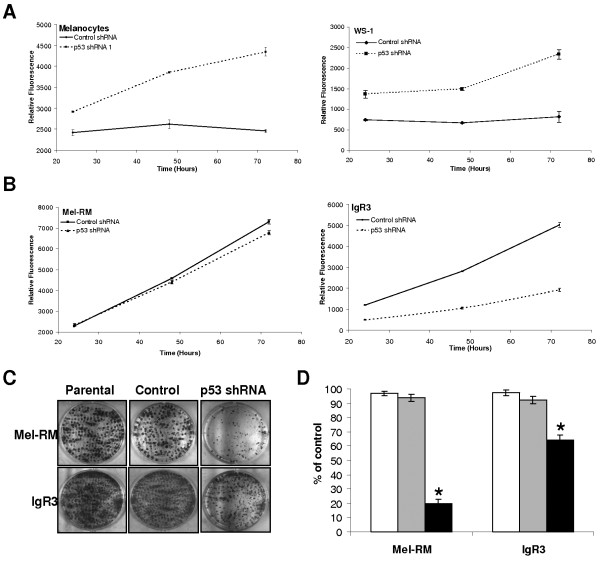
**Inhibition of P53 reduces proliferation in melanoma cells**. **(A) **Proliferation was analysed in melanocytes and WS-1 fibroblasts that had been stably transduced with *P53 *shRNA or control shRNA over a 72 hour period using the MTT assay. Results are represented as the mean ± SE of 3 experiments. **(B) **Proliferation was analysed in IgR3 and Mel-RM cells that had been stably transduced with *P53 *shRNA or control shRNA over a 72 hour period using the MTT assay. Results are represented as the mean ± SE of 3 experiments. **(C) and (D) **Proliferation was analysed by colony formation assay in IgR3 and Mel-RM cells that had been stably transduced with *P53 *shRNA (black bars) or control shRNA (grey bars) and compared to their parental counterparts (white bars). Representative results are shown in **(C) **and quantification of 3 independent experiments is shown in **(D) **with the number of colonies expressed as a percentage of the control shRNA transduced cell lines (mean ± SE). *p < 0.001 by students t-test.

Our gene expression data suggested that P53-dependent transcriptional control of target genes predominantly involved in cell cycle regulation was disrupted in melanoma when compared to melanocytes. Hence, to determine the effect of P53 on proliferation, we performed MTT and colony formation assays in cells transduced with either control shRNA or *P53 *shRNA. In melanocytes, the long-term reduction in P53 (greater than 4 weeks post-transduction) resulted in an overall increase in proliferation and an increased proliferation rate (measured as the gradient of each of the lines, 37.6 fold increase) when compared to cells that had been transduced with control shRNA (Figure 4A). The same effect was observed in WS-1 and HDF1314 fibroblasts (Figure 4A and data not shown). However, inhibition of P53 in melanoma cells reduced overall proliferation and the proliferation rate (-2.66 fold reduction in IgR3 cells and -1.13 fold reduction in Mel-RM), particularly in the IgR3 cell line, when compared to cells that had been transduced with control shRNA (Figure 4B). The long term growth potential of melanoma cell lines with P53 silenced was also significantly inhibited when compared to their control counterparts as determined by colony formation assays (Figure 4C and 4D). This suggests that the ability of wild-type P53 to inhibit cell growth in melanoma is disrupted when compared to normal cells, consistent with our gene expression analysis.

## Discussion

Although *P53 *is not commonly mutated in metastatic melanoma and can transcriptionally activate certain target genes in response to stress [5,17,18], its function is abnormal as reflected by a failure to induce cell cycle arrest and apoptosis [1,18]. In this study, we have examined the mRNA expression profile of known P53 target genes and regulators in a large number of melanoma metastases and cultured melanoma cell lines compared to normal melanocytes and fibroblasts to provide a global assessment of P53 functional aberration in melanoma.

P53 target genes involved in apoptosis and cell cycle regulation accounted for the large majority of transcripts altered in metastatic melanoma tumours and melanoma cell lines when compared to normal cells. In melanoma tissue extracts, P53 target genes involved in apoptosis were down-regulated compared to melanocytes; the exception being genes for *FLIP *(inhibitor of Caspase 8), *PMAIP1 (Noxa*, BH3 pro-apoptotic protein) and the *ATF3 *transcription factor (transcript variant 4 or deltaZip2) which can counteract transcriptional repression by full-length ATF3 [33]. Many cell cycle genes were also down-regulated, with the exception of the growth arrest and DNA damage inducible genes and a variant of *CDKN2A *(transcript variant 3). *Cyclin D2*, involved in cell cycle transition from G1 to S was also particularly high. These findings are supported by a recent study by Yu and colleagues who showed that benign nevi can be separated from melanomas on the basis of their P53 target gene expression profiles [34]. Of the 25 targets they identified as being consistently significantly different in two separate datasets of melanoma compared to nevi, almost half of the transcripts (9/25, 36%) were involved in cell cycle regulation or apoptosis further confirming our findings that these P53-dependent pathways are dysregulated in metastatic melanoma. However, in contrast to our analysis the majority of these transcripts showed increased mRNA expression in melanoma when compared to nevi and this discrepancy may be due to the imperfect comparison of melanocytes versus metastatic melanomas in our study [34].

In contrast to the studies on melanoma tissue, several cell cycle regulatory genes had significantly increased mRNA expression in melanoma lines reflecting their proliferative state. In particular, the cell cycle proteins BRCA1 and CHEK1 are capable of phosphorylating P53 and modulating its transcriptional activity [35-37]. Our previous studies (on the melanoma cell lines used in this study) have shown that P53 protein levels were much higher in melanoma cells than compared to melanocytes [16]. However, the expression of P53 target genes involved in apoptosis was generally much lower in melanoma cell lines compared to that in normal cells (for example *BAX *is normally increased by P53, but showed decreased expression in melanoma) and suggests that P53 signalling is aberrant in melanoma. Whether the increased expression of *BRCA1 *and *CHEK1 *may account for the increased expression of cell cycle genes and decreased expression of apoptotic target genes in melanoma is yet to be determined.

The studies on melanoma cell lines were surprising in that they revealed very few differences in P53 target gene expression between *P53 *null/mutant cell lines and those with wild-type *P53*, indicating that the constitutive regulation of these P53 target genes was not related to *P53 *status. Two of the genes that differed between these cell lines were, *CDKN2A *which encodes P14ARF and *BIRC5 *which encodes Survivin. These genes were also expressed significantly higher in melanoma cell lines when compared to normal cells. P14ARF enhances P53 functional activity by inhibiting MDM-2 mediated repression of P53 [38] and inactivation of *CDKN2A *is a common and critical event in the genesis of melanoma [39]. However, the *CDKN2A *locus was shown to be highly over-expressed in *P53 *null/mutant melanoma cells in this study, perhaps due to loss of feedback inhibition by P53 given that P53 can mediate transcriptional inhibition of *CDKN2A *[38]. Survivin is over-expressed in almost all human malignancies, including melanoma [40], consistent with its higher expression in melanoma cell lines observed in this study. Survivin is normally repressed by P53 in human melanocytes [41] as shown in the current study (Table 4), but was shown to be down-regulated in melanoma cell lines with null/mutant *P53 *when compared to those with wild-type *P53 *and was not altered by inhibition of P53 expression in melanoma, further suggesting aberrant transcriptional regulation of this target gene by P53 in melanoma.

To further examine the transcriptional regulation of P53 target genes, P53 expression was down-regulated by shRNA in melanocytes and two melanoma cell lines. Silencing of P53 resulted in significant changes in the mRNA expression of 19 P53 target genes in melanocytes and several of these target genes have previously been shown to be regulated by Nutlin activation of P53 in melanocytes, including CDKN1A and Survivin [32]. However, far fewer genes underwent significant changes in melanoma cell lines (IgR3-7 genes and Mel-RM-11 genes). Similar results were observed when whole genome gene expression was compared. These studies confirmed that the constitutive mRNA expression of many targets that were regulated by P53 in melanocytes were unaffected in melanoma, suggesting that P53 had lost the ability to regulate the expression of these transcripts in melanoma. In particular, there were several cell cycle genes whose transcription was increased by P53 inhibition in melanocytes (down-regulated in control shRNA cells) that showed a complete lack of P53-dependent regulation in melanoma cells. Given that many of these P53 target genes were over-expressed in melanoma when compared to normal cells (Table 2), this indicates that aberrant P53 signalling may play a role in the altered transcript expression of these genes and further suggests that P53-dependent pathways are disrupted in melanoma. Moreover, the ability of P53 to differentially regulate target genes involved in cell cycle function was confirmed in whole genome gene expression analysis, further emphasising this aberrant functional activity of P53.

Loss of P53 expression in mice and in human melanocytes has been shown to increase the proliferation and *in vivo *tumourigenicity, concordant with the role of P53 as a tumour suppressor [34,42]. An unexpected finding of this study was that inhibition of P53 function in melanocytes induced changes in gene expression profiles that were characteristic of melanoma cell lines expressing wild-type P53 and this resulted in increased proliferation in melanocytes, but not melanoma. These results further confirm that P53 function is aberrant in melanoma and imply that the disruption of P53-regulated pathways may be a contributing factor in the progression of melanoma. This study has identified several common P53-regulated targets that are pro-survival (*BIRC5, PLK2*) and/or are necessary for proper control of cell cycle progression (*CCNB2, CCNE2, CDC2, CDC25C, CDK6, CDKN2A*) that may be involved in this process. Moreover, the fact that inhibition of P53 resulted in decreased proliferation in melanoma suggests that the altered functional activity of P53 may promote tumour development and progression in melanoma, rather than suppress it. In this regard, there is evidence suggesting that P53 can protect cells from apoptosis. Almost 40 target genes, regulated by P53 have been shown to exert anti-apoptotic effects, suggesting that P53 can transcriptionally activate pro-survival pathways including those involved in the repair of damaged DNA, cell cycle arrest as well as those involved in the response to oxidative stress [43,44]. Furthermore, there have been reports demonstrating that cells lacking functional P53 were more susceptible to cell death induced by DNA-damaging agents [45-51]. The results presented herein suggest that P53 in melanoma, in the absence of any exogenous genotoxic stress, has not only lost its ability to control proliferation, but may indeed promote melanoma cell division. Although these results are in contrast to other reported studies on the role of p53 in melanoma progression [34,42], this challenges the notion that P53 is acting as a tumour suppressor in melanoma. In agreement with the current studies, it has been reported that P53 expression increases with melanoma progression and depth of tumour invasion, and that it is associated with worse prognostic features [5,32]. This was also noted by Terzian et al in their mouse model where P53 expression was shown to increase in TP-ras^0/+ ^mice in the progression from nevi to melanoma, where there was a tendency for rapidly growing melanomas to express high levels of P53 [32].

The reasons for the functional aberration of P53 in melanoma described in the current study are unclear. In particular instances, abnormal P53 function has been associated with a failure to up-regulate particular P53 target genes due to a variety of factors, including loss of adaptor proteins, deregulation of co-factors, or expression of proteins that inhibit the transcription of particular target genes [19,20,52,53]. However, with the exception of *BRCA1, CDKN2A *and *CHEK1 *which are known to enhance the cell cycle regulatory function of P53 [36,37], no other cofactors/regulators of P53 activity were altered in melanoma that could describe the lack of effect observed on P53 target genes as a result of P53 inhibition. Several factors may be involved in these changes. We have previously reported that small isoforms of P53, Δ40P53 and P53β, were highly expressed in melanoma cell lines when compared to normal cells and were associated with inhibition and enhancement respectively, of P53-dependent regulation of *P21 *and *PUMA *expression following treatment with Cisplatin [16]. P53β has also been shown to induce senescence [54]. The expression of these isoforms may modulate the constitutive P53-dependent regulation of these and other P53-dependent target genes. The activity of P53 in response to stress stimuli is tightly regulated by numerous post-translational modifications [55]. In particular, wild-type P53 in melanoma cells has been shown to be highly phosphorylated compared to normal cell lines and this would be expected to alter/impair its function in melanoma [18,56]. In addition, mono-methylation of P53 at Lys 370 has been shown to repress P53-mediated transcriptional regulation and apoptosis induction in H1299 cells [57]. Most recently, several studies have shown that the regulation of microRNAs miR-34a, miR-34b and miR-34c by P53 is vitally important for the regulation of P53-dependent apoptosis and cellular proliferation, with loss of mir-34a observed in several human cancers [58]. Whether these factors can account for the aberrant function of P53 in melanoma as observed in the current study awaits further investigation.

## Conclusions

In summary, this study has shown that not only is the mRNA expression of P53 target genes aberrant in melanoma, but that P53 has lost the ability to regulate its target genes, particularly those involved in cell cycle control and apoptosis. In fact the mRNA expression of these genes resembles that in melanocytes in which P53 has been knocked down by shRNA. The consequence of alterations in the P53 target genes observed in this study are altered growth/proliferation and a potential failure to elicit appropriate responses to apoptotic-inducing stimuli, such as chemotherapy. The gene expression results and studies on melanoma cell growth have provided provocative evidence that the P53 pathway in melanoma rather than acting as a tumour suppressor, may promote melanoma proliferation and progression. It remains now to identify the factor(s) responsible for the aberrant function of this transcription factor in melanoma.

## Competing interests

The authors declare that they have no competing interests.

## Authors' contributions

KAK carried out cell line studies, microarray analysis, real-time PCR confirmation and colony formation assays; she participated in study design/coordination and drafted the manuscript. NAB performed microarrays on melanoma cell lines and contributed to data analysis. AJC participated in microarray analysis on melanoma cell lines and performed western blots and proliferation assays. CFK, KAA and BTP performed microarrays on melanoma metastases and contributed to data analysis. LLS and HR made the lentiviral constructs and stably transfected cell lines. XDZ and RJS participated in study design and manuscript revision. PH conceived the study, participated in its design and coordination, and helped to draft the manuscript. All authors read and approved the final manuscript.

## Pre-publication history

The pre-publication history for this paper can be accessed here:

http://www.biomedcentral.com/1471-2407/11/203/prepub

## Supplementary Material

Additional file 1 **Supplementary Tables**. Contains Supplementary Table S1.
**Table S1: P53 target genes**. Probe ID, accession no., gene symbol and name of 290 probes used in analysis of P53 targets. Accompanying additional references are provided. Click here for file

Additional file 2 **Figure S1: Inhibition of P53 abolishes P21 expression**. The expression of P53 and P21 was analysed by western blotting in whole cell lysates from Mel-RM and IgR3 cells stably transduced with *P53 *shRNA or control shRNA and treated with CDDP (10 μg/ml) for the indicated times. The expression of GAPDH was determined to ensure equal loading. Results are representative of 3 independent experiments. Click here for file
