# Supplementary figures and images for: P53 in human melanoma fails to regulate target genes associated with apoptosis and the cell cycle and may contribute to proliferation
Source: BMC Cancer. 2011 May 27;11:203. doi: 10.1186/1471-2407-11-203 (PMC3120805; doi:10.1186/1471-2407-11-203)

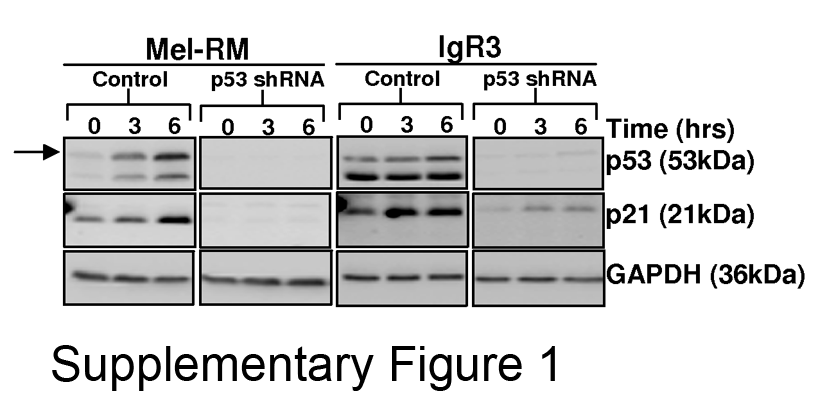

Supplement: Additional file 1 — Supplementary Tables. Contains Supplementary Table S1. Table S1: P53 target genes. Probe ID, accession no., gene symbol and name of 290 probes used in analysis of P53 targets. Accompanying additional references are provided. [file 1471-2407-11-203-S1.TIFF]
