# Supplementary material for: P53 in human melanoma fails to regulate target genes associated with apoptosis and the cell cycle and may contribute to proliferation
Source: BMC Cancer. 2011 May 27;11:203. doi: 10.1186/1471-2407-11-203 (PMC3120805; doi:10.1186/1471-2407-11-203)
Supplement: Additional file 2 — Figure S1: Inhibition of P53 abolishes P21 expression. The expression of P53 and P21 was analysed by western blotting in whole cell lysates from Mel-RM and IgR3 cells stably transduced with P53 shRNA or control shRNA and treated with CDDP (10 μg/ml) for the indicated times. The expression of GAPDH was determined to ensure equal loading. Results are representative of 3 independent experiments. [file 1471-2407-11-203-S2.PDF]

Supplementary Table

Supplementary Table 1: P53 targets. Description of the 290 probes used in the analysis.

Probe ID	Accession No.	Gene Symbol	Gene Name	Reference
7570326	NM_000024.4	*ADRB2*	Adrenergic, beta-2-, receptor	[1]
160343	NM_003748.2	*ALDH4A1*	Aldehyde dehydrogenase 4 family, member A1, transcript variant P5CDhL	[2]
1470427	NM_170726.1	*ALDH4A1*	Aldehyde dehydrogenase 4 family, member A1, transcript variant P5CDhS	[2]
6220360	NM_170726.1	*ALDH4A1*	Aldehyde dehydrogenase 4 family, member A1, transcript variant P5CDhS	[2]
1820324	NM_013229.2	*APAF1*	Apoptotic peptidase activating factor 1, transcript variant 5	[3-5]
2760082	NM_181869.1	*APAF1*	Apoptotic peptidase activating factor 1, transcript variant 5	[3-5]
6450669	NM_018685.2	*ANLN*	Anillin	[1]
5720722	NM_000038.3	*APC (FAP)*	Adenomatous polyposis coli	[4]
4780128	NM_001040619.1	*ATF3*	Activating transcription factor 3, transcript variant 4	[1]
6380735	NM_001030287.2	*ATF3*	Activating transcription factor 3, transcript variant 3	[1]
6560575	NM_001040619.1	*ATF3*	Activating transcription factor 3, transcript variant 4	[1]
580537	NM_000051.3	*ATM*	Ataxia telangiectasia mutated, transcript variant 1	[5-7]
4250008	NM_000051.3	*ATM*	Ataxia telangiectasia mutated, transcript variant 1	[5-7]
7330592	NM_138292.3	*ATM*	Ataxia telangiectasia mutated, transcript variant 2	[5-7]
3780364	XM_001131387.1	*ATR*	Ataxia telangiectasia and Rad3 related	[5-7]
4040286	NM_001184.2	*ATR*	Ataxia telangiectasia and Rad3 related	[5-7]
730575	XM_940726.1	*BAI1*	Brain-specific angiogenesis inhibitor 1, transcript variant 1	[2, 7, 8]
6220612	NM_001702.2	*BAI1*	Brain-specific angiogenesis inhibitor 1	[2, 7, 8]
6380711	NM_001702.2	*BAI1*	Brain-specific angiogenesis inhibitor 1	[2, 7, 8]
3520092	NM_138765.2	*BAX*	BCL2-associated X protein, transcript variant sigma (Bax zeta)	[2-5, 7-9]
4490538	NM_138764.2	*BAX*	BCL2-associated X protein, transcript variant epsilon (Bax zeta)	[2-5, 7-9]
7550110	NM_004324.3	*BAX*	BCL2-associated X protein, transcript variant beta (Bax zeta)	[2-5, 7-9]
3440364	NM_014417.2	*BBC3 (PUMA)*	BCL2 binding component 3	[3-5, 9]
3850576	NM_000657.2	*BCL2*	B-cell CLL/lymphoma 2, transcript variant beta	[4, 5]
3870474	NM_000657.2	*BCL2*	B-cell CLL/lymphoma 2, transcript variant beta	[4, 5]
4150201	NM_000633.2	*BCL2*	B-cell CLL/lymphoma 2, transcript variant alpha	[4, 5]
670673	NM_138578.1	*BCL2L1*	BCL2-like 1, transcript variant 1 (Bcl-xL)	[4, 5]
4290411	NM_138578.1	*BCL2L1*	BCL2-like 1, transcript variant 1 (Bcl-xL)	[4, 5]
2320719	NM_000623.2	*BDKRB2*	Bradykinin receptor B2	[9]
1190022	NM_197967.1	*BID*	BH3 interacting domain death agonist, transcript variant 3	[3-5, 7]
1440037	NM_197966.1	*BID*	BH3 interacting domain death agonist, transcript variant 1	[3-5, 7]
5270136	NM_197966.1	*BID*	BH3 interacting domain death agonist, transcript variant 1	[3-5, 7]
6280440	NM_001197.3	*BIK (BIP1)*	BCL2-interacting killer	[4]
1230682	NM_001168.2	*BIRC5*	Baculoviral IAP repeat-containing 5 (Survivin), transcript variant 1	[3, 5]
7550626	NM_001012271.1	*BIRC5*	Baculoviral IAP repeat-containing 5 (Survivin), transcript variant 3	[3, 5]
540411	NM_007304.2	*BRCA1*	Breast cancer 1, early onset, transcript variant BRCA1-delta11b	[10, 11]
2810465	NM_007299.2	*BRCA1*	Breast cancer 1, early onset, transcript variant BRCA1-delta15-17	[10, 11]
5820703	NM_007295.2	*BRCA1*	Breast cancer 1, early onset, transcript variant BRCA1b	[10, 11]
1010487	NM_006763.2	*BTG2*	B-cell translocation gene family member 2	[9]
2470315	NM_014477.2	*C20orf10*	Chromosome 20 open reading frame 10	[2, 4]
460382	NM_199141.1	*CARM1 (PRMT4)*	Coactivator-associated arginine methyltransferase 1	[12]
450491	NM_033294.2	*CASP1*	Caspase 1, transcript variant delta	[4]
6400053	NM_033292.2	*CASP1*	Caspase 1, transcript variant alpha	[4]
7050382	NM_033294.2	*CASP1*	Caspase 1, transcript variant delta	[4]

2630445	NM_032977.3	*CASP10*	Caspase 10, transcript variant B	[a]
7100301	NM_001230.4	*CASP10*	Caspase 10, transcript variant A	[a]
2640634	NR_000035.2	*CASP12*	Caspase 12	[a]
5670300	NM_012114.1	*CASP14*	Caspase 14	[a]
1440019	NM_032982.2	*CASP2*	Caspase 2, transcript variant 1	[13]
3360059	NM_032982.2	*CASP2*	Caspase 2, transcript variant 1	[13]
4570026	NM_032983.2	*CASP2*	Caspase 2, transcript variant 2	[13]
2260215	NM_032991.2	*CASP3*	Caspase 3, transcript variant beta	[7]
6330445	NM_004346.3	*CASP3*	Caspase 3, transcript variant alpha	[7]
3180164	NM_033307.2	*CASP4*	Caspase 4, transcript variant delta	[a]
3850021	NM_001225.3	*CASP4*	Caspase 4, transcript variant gamma	[a]
4830242	NM_001225.3	*CASP4*	Caspase 4, transcript variant alpha	[a]
6100136	NM_033306.2	*CASP4*	Caspase 4, transcript variant gamma	[a]
3390121	NM_004347.1	*CASP5*	Caspase 5	[a]
2320730	NM_032992.2	*CASP6*	Caspase 6, transcript variant beta	[3-5]
4730577	NM_001226.3	*CASP6*	Caspase 6, transcript variant alpha	[3-5]
5550709	NM_001226.3	*CASP6*	Caspase 6, transcript variant alpha	[3-5]
5290465	NM_033339.3	*CASP7*	Caspase 7, transcript variant gamma	[a]
6370441	NM_033338.4	*CASP7*	Caspase 7, transcript variant delta	[a]
7650324	NM_033340.2	*CASP7*	Caspase 7, transcript variant beta	[a]
290592	NM_033356.3	*CASP8*	Caspase 8, transcript variant C	[4]
460424	NM_001228.4	*CASP8*	Caspase 8, transcript variant A	[4]
1050092	NM_033355.3	*CASP8*	Caspase 8, transcript variant B	[4]
2650750	NM_001080125.1	*CASP8*	Caspase 8, transcript variant G	[4]
1050524	NM_012115.2	*CASP8AP2*	CASP8 associated protein 2	[a]
2340041	NM_001229.2	*CASP9*	Caspase 9, transcript variant alpha	[7]
4280735	NM_032996.1	*CASP9*	Caspase 9, transcript variant alpha	[7]
4590040	NM_031966.2	*CCNB1*	Cyclin B1	[7]
5360070	NM_004701.2	*CCNB2*	Cyclin B2	[14]
1770239	NM_033031.2	*CCNB3*	Cyclin B3, transcript variant 3	[14]
3310379	NM_033670.2	*CCNB3*	Cyclin B3, transcript variant 1	[14]
6520133	NM_033031.2	*CCNB3*	Cyclin B3, transcript variant 3	[14]
5820601	NM_053056.2	*CCND1*	Cyclin D1	[7]
3460520	NM_001759.2	*CCND2*	Cyclin D2	[14]
6380370	NM_001760.2	*CCND3*	Cyclin D3	[14]
3990026	NM_001238.1	*CCNE1*	Cyclin E1, transcript variant 1	[7]
6590093	NM_001238.1	*CCNE1*	Cyclin E1, transcript variant 1	[7]
1450041	NM_057749.1	*CCNE2*	Cyclin E2	[14]
4760154	NM_057735.1	*CCNE2*	Cyclin E2, transcript variant 2	[14]
3170110	NM_199246.1	*CCNG1*	Cyclin G1, transcript variant 2	[9]
4570520	NM_199246.1	*CCNG1*	Cyclin G1, transcript variant 2	[9]
5360672	NM_004354.1	*CCNG2*	Cyclin G2	[14]
2340768	NM_001024844.1	*CD82*	CD82 molecule, transcript variant 2	[14]
5130136	NM_002231.3	*CD82*	CD82 antigen, transcript variant 1	[14]
1050706	NM_001786.2	*CDC2*	Cell division cycle 2, transcript variant 2	[7]
2760603	NM_001786.2	*CDC2*	Cell division cycle 2, transcript variant 1	[7]
20605	NM_001790.3	*CDC25C*	Cell division cycle 25 homolog C, transcript variant 1	[9]
3460152	NM_022809.2	*CDC25C*	Cell division cycle 25 homolog C, transcript variant 2	[9]
4200451	NM_001790.3	*CDC25C*	Cell division cycle 25 homolog C, transcript variant 1	[9]
4230201	NM_000389.2	*CDKN1A*	Cyclin-dependent kinase inhibitor 1A, transcript variant 1	[1, 2, 5, 7-9]
5290475	NM_078467.1	*CDKN1A*	Cyclin-dependent kinase inhibitor 1A, transcript variant 2	[1, 2, 5, 7-9]
5130671	NM_058197.3	*CDKN2A*	Cyclin-dependent kinase inhibitor 2A, transcript variant 3	[7, 12]
5550671	NM_058195.2	*CDKN2A*	Cyclin-dependent kinase inhibitor 2A, transcript variant 4	[7, 12]
6620014	NM_000077.3	*CDKN2A*	Cyclin-dependent kinase inhibitor 2A, transcript variant 3	[7, 12]
2350286	NM_001798.2	*CDK2*	Cyclin-dependent kinase 2, transcript variant 1	[7]
4590064	NM_001798.2	*CDK2*	Cyclin-dependent kinase 2, transcript variant 1	[7]
7380110	NM_000075.2	*CDK4*	Cyclin-dependent kinase 4	[14]
670286	NM_001259.5	*CDK6*	Cyclin-dependent kinase 6	[14]
1240593	NM_003879.3	*CFLAR*	CASP8 and FADD-like apoptosis regulator (c-FLIP)	[4]
7200270	NM_001274.3	*CHEK1*	CHK1 checkpoint homolog	[7, 12]
5260685	NM_007194.3	*CHEK2*	CHK2 checkpoint homolog, transcript variant 1	[7, 12]
6510079	NM_001005735.1	*CHEK2*	CHK2 checkpoint homolog, transcript variant 3	[7, 12]

6560438	NM_004380.2	*CREBBP (CBP)*	CREB binding protein, transcript variant 1	[7, 12]
6840148	NM_001079846.1	*CREBBP (CBP)*	CREB binding protein	[7, 12]
4280487	NM_003296.1	*CRISP2*	Cysteine-rich secretory protein 2	[7]
3850010	NM_001889.2	*CRYZ*	Crystallin, zeta	[9]
1110092	NM_001909.3	*CTSD*	Cathepsin D	[4]
630521	NM_002996.3	*CX3CL1*	Chemokine (C-X3-C motif) ligand 1	[2, 9]
6200554	NM_018947.4	*CYCS*	Cytochrome c	[7]
3190438	NM_001037333.1	*CYFIP2 (PIR121)*	Cytoplasmic FMR1 interacting protein 2, transcript variant 1	[15]
3520020	NM_001037333.1	*CYFIP2 (PIR121)*	Cytoplasmic FMR1 interacting protein 2, transcript variant 1	[15]
6100768	NM_014376.2	*CYFIP2 (PIR121)*	Cytoplasmic FMR1 interacting protein 2, transcript variant 3	[15]
2000482	NM_000107.1	*DDB2*	Damage-specific DNA binding protein 2	[1]
3190148	NM_019058.2	*DDIT4 (REDD1)*	DNA-damage-inducible transcript 4	[4]
4880609	NM_012242.2	*DKK1*	Dickkopf homolog 1	[1, 9]
3450719	NM_001402.5	*EEF1A1*	Eukaryotic translation elongation factor 1 alpha 1	[4, 9]
3850121	NM_001402.5	*EEF1A1*	Eukaryotic translation elongation factor 1 alpha 1	[4, 9]
1050671	NM_005228.3	*EGFR (ERBB)*	Epidermal growth factor receptor, transcript variant 1	[7, 9]
3390538	NM_201284.1	*EGFR (ERBB)*	Epidermal growth factor receptor, transcript variant 4	[7, 9]
4070019	NM_201282.1	*EGFR (ERBB)*	Epidermal growth factor receptor, transcript variant 2	[7, 9]
6330307	NM_201282.1	*EGFR (ERBB1)*	Epidermal growth factor receptor, transcript variant 2	[7, 9]
770541	NM_001007277.1	*EI24 (PIG8)*	Etoposide induced 2.4 mRNA, transcript variant 2	[5]
5310707	NM_004879.3	*EI24 (PIG8)*	Etoposide induced 2.4 mRNA, transcript variant 1	[5]
6510332	NM_004431.2	*EPHA2 (ECK)*	EPH receptor A2	[1, 4]
460438	NM_152872.1	*FAS*	TNF receptor superfamily, member 6, transcript variant 2	[3-5, 7-9]
3420026	NM_152877.1	*FAS*	TNF receptor superfamily, member 6, transcript variant 7	[3-5, 7-9]
1400441	NM_024417.1	*FDXR (ADXR)*	Ferredoxin reductase, transcript variant 1	[3-5]
2900056	NM_004110.3	*FDXR (ADXR)*	Ferredoxin reductase, transcript variant 2	[3-5]
990288	NM_201555.1	*FHL2*	Four and a half LIM domains 2, transcript variant 2	[4]
6110025	NM_201557.2	*FHL2*	Four and a half LIM domains 2, transcript variant 4	[4]
7040681	NM_201555.1	*FHL2*	Four and a half LIM domains 2, transcript variant 2	[4]
7610632	NM_201557.2	*FHL2*	Four and a half LIM domains 2, transcript variant 4	[4]
4280017	NM_005252.2	*FOS*	v-fos FBJ murine osteosarcoma viral oncogene homolog	[4]
4880673	NM_001924.2	*GADD45A*	Growth arrest and DNA-damage-inducible, alpha	[2, 7-9]
4920110	NM_015675.2	*GADD45B*	Growth arrest and DNA-damage-inducible, beta	[14, 16]
5570114	NM_006705.2	*GADD45G*	Growth arrest and DNA-damage-inducible, gamma	[14]
5090671	NM_004864.1	*GDF15*	Growth differentiation factor 15	[9]
4220689	NM_002066.1	*GML*	GPI anchored molecule like protein	[2, 4, 7, 9]
60647	NM_201397.1	*GPX1*	Glutathione peroxidase 1, transcript variant 2	[4]
5720600	NM_201397.1	*GPX1*	Glutathione peroxidase 1, transcript variant 2	[4]
6980382	NM_016426.4	*GTSE1*	G-2 and S-phase expressed 1	[7, 14]
780634	NM_001010934.1	*HGF*	Hepatocyte growth factor, transcript variant 5	[9]
2340292	NM_001010932.1	*HGF*	Hepatocyte growth factor, transcript variant 3	[9]
3130451	NM_000601.4	*HGF*	Hepatocyte growth factor, transcript variant 1	[9]
10332	NM_022740.2	*HIPK2*	Homeodomain interacting protein kinase 2	[12]
430672	NM_153201.1	*HSPA8*	Heat shock 70kDa protein 8, transcript variant 2	[9]
2650619	NM_006597.3	*HSPA8*	Heat shock 70kDa protein 8, transcript variant 1	[9]
7200608	NM_001540.2	*HSPB1*	Heat shock 27kDa protein 1	[4]
1190367	NM_003897.3	*IER3*	Immediate early response 3	[9]
3310400	NM_000618.2	*IGF1*	Insulin-like growth factor 1	[14]
6590132	NM_000598.4	*IGFBP3*	Insulin-like growth factor binding protein 3, transcript variant 2	[4, 7, 9]
6840372	NM_001013398.1	*IGFBP3*	Insulin-like growth factor binding protein 3, transcript variant 1	[4, 7, 9]
2230431	NM_002200.3	*IRF5*	Interferon regulatory factor 5, transcript variant 1	[2]
2630554	NM_152405.2	*JMY*	Junction-mediating and regulatory protein	[12]
2650056	XM_001133677.1	*LOC729264*	PREDICTED: Similar to TP53TG3 protein, transcript variant 2	**a**
580242	NM_018494.3	*LRDD (PIDD)*	Leucine-rich repeats and death domain containing, transcript variant 2	[4, 5, 8, 9]
5130022	NM_145887.2	*LRDD (PIDD)*	Leucine-rich repeats and death domain containing, transcript variant 3	[4, 5, 8, 9]
6550403	NM_018494.3	*LRDD (PIDD)*	Leucine-rich repeats and death domain containing, transcript variant 2	[4, 5, 8, 9]

1740082	NM_020128.1	*MDM1*	Mdm4, transformed 3T3 cell double minute 1, transcript variant 2	[17]
3830114	NM_020128.1	*MDM1*	Mdm4, transformed 3T3 cell double minute 1, transcript variant 2	[17]
4730546	NM_017440.2	*MDM1*	Mdm4, transformed 3T3 cell double minute 1, transcript variant 1	[17]
630600	NM_002392.2	*MDM2 (HDM2)*	Mdm2 p53 binding protein homolog, transcript variant MDM2	[2, 5, 7]
6840438	NM_006879.2	*MDM2 (HDM2)*	Mdm2 p53 binding protein homolog, transcript variant MDM2b	[2, 5, 7]
5420471	NM_002393.2	*MDM4*	Mdm4, transformed 3T3 cell double minute 4	[12]
4200504	NM_000245.2	*MET*	Met proto-oncogene (hepatocyte growth factor receptor)	[9]
510736	NM_004530.2	*MMP2*	Matrix metallopeptidase 2	[9]
4260224	NM_006792.2	*MORF4*	Mortality factor 4	[2]
2480195	NM_000603.3	*NOS3*	Nitric oxide synthase 3	[9]
6020192	NM_199185.2	*NPM1*	Nucleophosmin, transcript variant 2	[12]
4070017	NM_002539.1	*ODC1*	Ornithine decarboxylase 1	[9]
3170661	NM_005446.2	*P2RXL1*	Purinergic receptor P2X-like 1, orphan receptor	[7, 9]
3710543	NM_022112.1	*P53AIP1*	p53-regulated apoptosis-inducing protein 1	[2-5, 8, 9]
630274	NM_033009.1	*PCBP4 (LIP4)*	Poly(rC) binding protein 4, transcript variant 2	[9]
2850598	NM_033008.1	*PCBP4 (LIP4)*	Poly(rC) binding protein 4, transcript variant 3	[9]
4050563	NM_020418.2	*PCBP4 (LIP4)*	Poly(rC) binding protein 4, transcript variant 1	[9]
5690743	NM_020418.2	*PCBP4 (LIP4)*	Poly(rC) binding protein 4, transcript variant 1	[9]
1410524	NM_002592.2	*PCNA*	Proliferating cell nuclear antigen, transcript variant 1	[7, 9]
2510348	NM_182649.1	*PCNA*	Proliferating cell nuclear antigen, transcript variant 2	[7, 9]
6900079	NM_182649.1	*PCNA*	Proliferating cell nuclear antigen, transcript variant 2	[7, 9]
3850468	NM_006210.1	*PEG3*	Paternally expressed 3	[9]
4040754	NM_022121.2	*PERP*	PERP, TP53 apoptosis effector	[3-5, 8]
1470753	NM_181523.1	*PIK3R1*	Phosphoinositide-3-kinase, regulatory subunit 1 (alpha), transcript variant 1	[4]
1510209	NM_181504.2	*PIK3R1*	Phosphoinositide-3-kinase, regulatory subunit 1 (alpha) , transcript variant 2	[4]
4290450	NM_181524.1	*PIK3R1*	Phosphoinositide-3-kinase, regulatory subunit 1 (alpha), transcript variant 3	[4]
4610626	NM_181523.1	*PIK3R1*	Phosphoinositide-3-kinase, regulatory subunit 1 (alpha), transcript variant 1	[4]
6520630	NM_181504.2	*PIK3R1*	Phosphoinositide-3-kinase, regulatory subunit 1 (alpha), transcript variant 2	[4]
1740746	NM_006622.2	*PLK2*	Polo-like kinase 2	[9]
2750367	NM_021127.1	*PMAIP1 (NOXA)*	Phorbol-12-myristate-13-acetate-induced protein 1	[2-5, 8]
520070	NM_033246.2	*PML*	Promyelocytic leukemia, transcript variant 7	[7, 12]
1230091	NM_033238.2	*PML*	Promyelocytic leukemia, transcript variant 1	[7, 12]
2070682	NM_033239.2	*PML*	Promyelocytic leukemia, transcript variant 7	[7, 12]
3180452	NM_033239.2	*PML*	PREDICTED: Promyelocytic leukemia, transcript variant 13	[7, 12]
7560546	NM_033240.2	*PML*	Promyelocytic leukemia, transcript variant 2	[7, 12]
3420228	NM_015316.2	*PPP1R13B (ASPP1)*	Protein phosphatase 1, regulatory (inhibitor) subunit 13B	[12]
1240176	NM_006663.2	*PPP1R13L (iASPP)*	Protein phosphatase 1, regulatory (inhibitor) subunit 13 like	[12]
5690554	NM_003620.2	*PPM1D (WIP1)*	Protein phosphatase 1D magnesium-dependent, delta	[7]
2360128	NM_006093.2	*PRG3*	Proteoglycan 3	[4]
1170288	NM_198319.2	*PRMT1*	Protein arginine methyltransferase 1, transcript variant 2	[12]
3460669	NM_198318.1	*PRMT1*	Protein arginine methyltransferase 1, transcript variant 3	[12]
6400138	NM_198319.2	*PRMT1*	Protein arginine methyltransferase 1, transcript variant 2	[12]
1470392	NM_001535.2	*PRMT2*	Protein arginine methyltransferase 2, transcript variant 2	[a]
2640161	NM_206962.1	*PRMT2*	Protein arginine methyltransferase 2, transcript variant 1	[a]
3850615	NM_001535.2	*PRMT2*	Protein arginine methyltransferase 2, transcript variant 1	[a]
3850520	NM_001039619.1	*PRMT5*	Protein arginine methyltransferase 5, transcript variant 2	[18]
5700520	NM_006109.3	*PRMT5*	Protein arginine methyltransferase 5	[18]
7200707	NM_018137.1	*PRMT6*	Protein arginine methyltransferase 6	[a]
2100402	NM_019023.1	*PRMT7*	Protein arginine methyltransferase 7	[a]
1190477	NM_019854.3	*PRMT8*	Protein arginine methyltransferase 8	[a]
5860689	NM_016335.2	*PRODH (PIG6)*	Proline dehydrogenase 1	[4, 5]
6840072	NM_000314.4	*PTEN*	Phosphatase and tensin homolog	[3, 5, 9, 14]
1820632	NM_000963.1	*PTGS2 (COX2)*	Prostaglandin-endoperoxide synthase 2 (Cyclooxygenase)	[4]
6280474	NM_000321.2	*RB1*	Retinoblastoma 1	[9]

70630	NM_182757.2	*RNF144B*	Ring finger 144B	[2]
270445	NM_001008925.1	*RCHY1*	Ring finger and CHY zinc finger domain containing 1, transcript variant 1	[19]
4220220	NM_001009922.1	*RCHY1*	Ring finger and CHY zinc finger domain containing 1, transcript variant 3	[19]
5550414	NM_019845.2	*RPRM*	Reprimo	[2, 8]
130639	NM_022457.5	*RFWD2*	Ring finger and WD repeat domain 2, transcript variant 1	
2940243	NM_022457.5	*RFWD2*	Ring finger and WD repeat domain 2, transcript variant 1	[19]
7570066	NM_022457.5	*RFWD2*	Ring finger and WD repeat domain 2, transcript variant 1	[19]
510731	NM_001034.1	*RRM2*	Ribonucleotide reductase M2 polypeptide	[20]
1030639	NM_015713.3	*RRM2B*	Ribonucleotide reductase M2 B (TP53 inducible, p53R2)	[2, 9]
2970017	NM_005978.3	*S100A2*	S100 calcium binding protein A2	[9]
2190278	NM_182826.1	*SCARA3*	Scavenger receptor class A, member 3, transcript variant 2	[2, 9]
6250349	NM_182826.1	*SCARA3*	Scavenger receptor class A, member 3, transcript variant 2	[2, 9]
6420630	NM_016240.2	*SCARA3*	Scavenger receptor class A, member 3, transcript variant 1	[2, 9]
2140128	NM_005063.4	*SCD*	Stearoyl-CoA desaturase	[9]
4810468	NM_003005.2	*SELP*	Selectin P	[9]
430181	NM_001005914.1	*SEMA3B*	Semaphorin 3B, transcript variant 2	[2]
4220433	NM_001005914.1	*SEMA3B*	Semaphorin 3B , transcript variant 2	[2]
6290707	NM_002639.3	*SERPINB5*	Serpin peptidase inhibitor, clade B, member 5 (Maspin)	[2, 8, 9]
770408	NM_000602.1	*SERPINE1 (PAI1)*	Serpin peptidase inhibitor, clade E, member 1	[7, 9]
1240553	NM_014454.1	*SESN1*	Sestrin 1	[9, 21, 22]
6650630	NM_031459.3	*SESN2*	Sestrin 2	[9, 21, 22]
2480192	NM_144665.2	*SESN3*	Sestrin 3	[14]
3710040	NM_006142.3	*SFN*	Stratifin	[2, 7-9]
160731	NM_016479.3	*SHISA5*	Shisa homolog 5 (Xenopus laevis) (Scotin)	[4, 5, 8]
3830408	NM_001006610.1	*SIAH1*	Seven in absentia homolog 1, transcript variant 2	[7]
4670148	NM_003031.3	*SIAH1*	Seven in absentia homolog 1 , transcript variant 1	[7]
7570537	NM_001006610.1	*SIAH1*	Seven in absentia homolog 1, transcript variant 2	[7]
4670487	NM_021709.1	*SIVA*	CD27-binding (Siva) protein, transcript variant 2	[23]
5550673	NM_006427.2	*SIVA*	CD27-binding (Siva) protein, transcript variant 1	[23]
1740136	NM_018976.3	*SLC38A2*	Solute carrier family 38, member 2	[9]
6330730	NM_003068.3	*SNAI2 (SLUG)*	Snail homolog 2	[24]
2260176	NM_152546.1	*SRFBP1*	Serum response factor binding protein 1	[12]
7570026	NM_005862.2	*STAG1*	Stromal antigen 1	[2]
620703	NM_182915.2	*STEAP3*	STEAP family member 3 , transcript variant 1	[25]
1300762	NM_018234.2	*STEAP3*	STEAP family member 3, transcript variant 2	[25]
1470097	NM_018234.2	*STEAP3*	STEAP family member 3, transcript variant 2	[25]
4200672	NM_007178.3	*STRAP*	Serine/threonine kinase receptor associated protein	[12]
7330392	NM_000593.5	*TAP1*	Transporter 1, ATP-binding cassette, sub-family B	[9]
6660435	NM_003236.1	*TGFA*	Transforming growth factor, alpha	[7]
5810685	NM_003246.2	*THBS1 (TSP1)*	Thrombospondin 1	[5, 7, 8]
2600164	NM_003247.2	*THBS2 (TSP2)*	Thrombospondin 2	[9]
1050762	NM_003844.2	*TNFRSF10A*	Tumor necrosis factor receptor superfamily, member 10a	[5]
1710014	NM_147187.1	*TNFRSF10B*	Tumor necrosis factor receptor superfamily, member 10b, transcript variant 2 (TRAIL-R2)	[2-5, 7-9]
2360095	NM_003842.3	*TNFRSF10B*	Tumor necrosis factor receptor superfamily, member 10b, transcript variant 1 (TRAIL-R2)	[2-5, 7-9]
2600463	NM_003842.3	*TNFRSF10B*	Tumor necrosis factor receptor superfamily, member 10b, transcript variant 1 (TRAIL-R2)	[2-5, 7-9]
2260746	NM_003841.2	*TNFRSF10C*	Tumor necrosis factor receptor superfamily, member 10c (TRAIL-R3)	[4]
830113	NM_003840.3	*TNFRSF10D*	Tumor necrosis factor receptor superfamily, member 10d (TRAIL-R4)	[4]
450376	NM_001077183.1	*TSC2*	Tuberous sclerosis 2, transcript variant 2	[26]
3370497	NM_000548.3	*TSC2*	Tuberous sclerosis 2, transcript variant 1	[26]
5670544	NM_005802.2	*TOPORS*	Topoisomerase I binding, arginine/serine-rich	[12]
4180500	NM_000546.3	*TP53*	Tumor protein p53	[14]
610475	NM_005657.1	*TP53BP1*	Tumor protein p53 binding protein 1	[12]
150095	NM_001031685.2	*TP53BP2*	Tumor protein p53 binding protein 2, transcript variant 1	[5, 12]
5570201	NM_005426.2	*TP53BP2*	Tumor protein p53 binding protein 2, transcript variant 2	[5, 12]
70440	NM_006034.2	*TP53I11 (PIG11)*	Tumor protein p53 inducible protein 11	[5, 7]
6350376	NM_006034.3	*TP53I11 (PIG11)*	Tumor protein p53 inducible protein 11	[5, 7]
1010647	NM_138349.2	*TP53I13 (DSCP1)*	Tumor protein p53 inducible protein 13	[5, 7]
1260020	NM_147184.1	*TP53I3 (PIG3)*	Tumor protein p53 inducible protein 3, transcript variant 2	[1, 4, 5, 7]

4640341	NM_004881.2	*TP53I3 (PIG3)*	Tumor protein p53 inducible protein 3, transcript variant 1	[1, 4, 5, 7]
5420538	NM_033285.2	*TP53INP1*	Tumor protein p53 inducible nuclear protein 1	[2-4]
5360392	NM_021202.1	*TP53INP2*	Tumor protein p53 inducible nuclear protein 2	[17]
2320037	NM_033550.3	*TP53RK*	TP53 regulating kinase	[17]
6060376	NM_016212.2	*TP53TG3*	TP53 target gene 3	[17]
6110041	NM_005427.1	*TP73*	Tumor protein p73	[5, 7]
6060131	NM_003722.3	*TP73L (TP63)*	Tumor protein p73-like	[5]
2650477	NM_004295.3	*TRAF4 (CART1)*	TNF receptor-associated factor 4	[4, 7]
3610301	NM_004295.3	*TRAF4 (CART1)*	TNF receptor-associated factor 4	[4, 7]
5700068	NM_001001188.3	*TRPM2*	Transient receptor potential cation channel, subfamily M, member 2, transcript variant S	[9]
7320497	NM_001001188.3	*TRPM2*	Transient receptor potential cation channel, subfamily M, member 2, transcript variant S	[9]
380369	NM_003496.1	*TRRAP*	Transformation/transcription domain-associated protein	[12]
1300411	NM_170744.2	*UNC5B*	Unc-5 homolog B (C. elegans) (p53RDL1)	[2, 4]
4880070	NM_001017535.1	*VDR*	Vitamin D receptor, transcript variant 2	
4150053	NM_004559.3	*YBX1*	Y box binding protein 1	[12, 28]
6560601	NM_152240.1	*ZMAT3 (WIG1)*	Zinc finger, matrin type 3, transcript variant 2	[4, 7]

[a]Related gene family member is regulated by p53.

Supplementary References

1. Yang G, Zhang G, Pittelkow MR, Ramoni M, Tsao H: **Expression profiling of UVB response in melanocytes identifies a set of p53-target genes.** *J Invest Dermatol* 2006, **126:**2490-2506.
2. Nakamura Y: **Isolation of p53-target genes and their functional analysis.** *Cancer Sci* 2004, **95:**7-11.
3. Fridman JS, Lowe SW: **Control of apoptosis by p53.** *Oncogene* 2003, **22:**9030-9040.
4. Harms K, Nozell S, Chen X: **The common and distinct target genes of the p53 family transcription factors.** *Cell Mol Life Sci* 2004, **61:**822-842.
5. Yu J, Zhang L: **The transcriptional targets of p53 in apoptosis control.** *Biochem Biophys Res Commun* 2005, **331:**851-858.
6. Efeyan A, Serrano M: **p53: guardian of the genome and policeman of the oncogenes.** *Cell Cycle* 2007, **6:**1006-1010.
7. Harris SL, Levine AJ: **The p53 pathway: positive and negative feedback loops.** *Oncogene* 2005, **24:**2899-2908.
8. Vogelstein B, Lane D, Levine AJ: **Surfing the p53 network.** *Nature* 2000, **408:**307-310.
9. **p53 Knowledgebase** [http://p53.bii.a-star.edu.sg/index.php]
10. Fabbro M, Savage K, Hobson K, Deans AJ, Powell SN, McArthur GA, Khanna KK: **BRCA1-BARD1 complexes are required for p53Ser-15 phosphorylation and a G1/S arrest following ionizing radiation-induced DNA damage.** *J Biol Chem* 2004, **279:**31251-31258.
11. Foray N, Marot D, Gabriel A, Randrianarison V, Carr AM, Perricaudet M, Ashworth A, Jeggo P: **A subset of ATM- and ATR-dependent phosphorylation events requires the BRCA1 protein.** *Embo J* 2003, **22:**2860-2871.
12. Coutts AS, La Thangue NB: **The p53 response: emerging levels of co-factor complexity.** *Biochem Biophys Res Commun* 2005, **331:**778-785.
13. Seth R, Yang C, Kaushal V, Shah SV, Kaushal GP: **p53-dependent caspase-2 activation in mitochondrial release of apoptosis-inducing factor and its role in renal tubular epithelial cell injury.** *J Biol Chem* 2005, **280:**31230-31239.
14. **Kyoto Encyclopedia of Genes and Genomes- p53 signaling pathway** [http://www.genome.jp/dbget-bin/www_bget?ko04115]
15. Jackson RS, 2nd, Cho YJ, Stein S, Liang P: **CYFIP2, a direct p53 target, is leptomycin-B sensitive.** *Cell Cycle* 2007, **6:**95-103.
16. Lambert JM, Moshfegh A, Hainaut P, Wiman KG, Bykov VJ: **Mutant p53 reactivation by PRIMA-1(MET) induces multiple signaling pathways converging on apoptosis.** *Oncogene* 2009, **29:**1329-1338.
17. **SOURCE** [http://smd.stanford.edu/cgi-bin/source/sourceSearch]
18. Jansson M, Durant ST, Cho EC, Sheahan S, Edelmann M, Kessler B, La Thangue NB: **Arginine methylation regulates the p53 response.** *Nat Cell Biol* 2008, **10:**1431-1439.
19. Corcoran CA, Huang Y, Sheikh MS: **The p53 paddy wagon: COP1, Pirh2 and MDM2 are found resisting apoptosis and growth arrest.** *Cancer Biol Ther* 2004, **3:**721-725.
20. Xue L, Zhou B, Liu X, Qiu W, Jin Z, Yen Y: **Wild-type p53 regulates human ribonucleotide reductase by protein-protein interaction with p53R2 as well as hRRM2 subunits.** *Cancer Res* 2003, **63:**980-986.
21. Budanov AV, Shoshani T, Faerman A, Zelin E, Kamer I, Kalinski H, Gorodin S, Fishman A, Chajut A, Einat P, et al: **Identification of a novel stress-responsive gene Hi95 involved in regulation of cell viability.** *Oncogene* 2002, **21:**6017-6031.
22. Velasco-Miguel S, Buckbinder L, Jean P, Gelbert L, Talbott R, Laidlaw J, Seizinger B, Kley N: **PA26, a novel target of the p53 tumor suppressor and member of the GADD family of DNA damage and growth arrest inducible genes.** *Oncogene* 1999, **18:**127-137.
23. Jacobs SB, Basak S, Murray JI, Pathak N, Attardi LD: **Siva is an apoptosis-selective p53 target gene important for neuronal cell death.** *Cell Death Differ* 2007, **14:**1374-1385.
24. Wu WS, Heinrichs S, Xu D, Garrison SP, Zambetti GP, Adams JM, Look AT: **Slug antagonizes p53-mediated apoptosis of hematopoietic progenitors by repressing puma.** *Cell* 2005, **123:**641-653.

25. Passer BJ, Nancy-Portebois V, Amzallag N, Prieur S, Cans C, Roborel de Climens A, Fiucci G, Bouvard V, Tuynder M, Susini L, et al: **The p53-inducible TSAP6 gene product regulates apoptosis and the cell cycle and interacts with Nix and the Myt1 kinase.** *Proc Natl Acad Sci U S A* 2003, **100:**2284-2289.
26. Feng Z, Hu W, de Stanchina E, Teresky AK, Jin S, Lowe S, Levine AJ: **The regulation of AMPK beta1, TSC2, and PTEN expression by p53: stress, cell and tissue specificity, and the role of these gene products in modulating the IGF-1-AKT-mTOR pathways.** *Cancer Res* 2007, **67:**3043-3053.
27. Maruyama R, Aoki F, Toyota M, Sasaki Y, Akashi H, Mita H, Suzuki H, Akino K, Ohe-Toyota M, Maruyama Y, et al: **Comparative genome analysis identifies the vitamin D receptor gene as a direct target of p53-mediated transcriptional activation.** *Cancer Res* 2006, **66:**4574-4583.
28. Schittek B, Psenner K, Sauer B, Meier F, Iftner T, Garbe C: **The increased expression of Y box-binding protein 1 in melanoma stimulates proliferation and tumor invasion, antagonizes apoptosis and enhances chemoresistance.** *Int J Cancer* 2007, **120:**2110-2118.
